# Assessing first-line treatment for advanced EGFR-mutated NSCLC in diverse clinicopathological subgroups: a systematic review and network meta-analysis

**DOI:** 10.1186/s12885-025-15236-z

**Published:** 2025-11-14

**Authors:** Ting Mei, Ting Wang, Qinghua Zhou

**Affiliations:** 1 Lung Cancer Center/Lung Cancer Institute, West China Hospital, Sichuan University, Chengdu, 610000 China; 2 Department of Medical Oncology, Cancer Center, West China Hospital, Sichuan University, Chengdu, Sichuan China

**Keywords:** Epidermal growth factor receptor, Efficacy, First-line treatment, Network meta-analysis, Non-small cell lung cancer

## Abstract

**Background:**

This network meta-analysis (NMA) aimed to indicate the most effective first-line therapeutic options for advanced EGFR-mutated NSCLC, particularly considering their specific clinicopathological characteristics.

**Methods:**

Articles in the EMBASE, Cochrane Library, PubMed, and Web of Science databases and conference abstracts published as of December 2023, were searched to obtain data from randomized controlled trials (RCTs) of the first-line treatment of advanced EGFR-mutated NSCLC cases with EGFR-TKIs alone or together with other agents.

**Results:**

37 RCTs including 24 treatment regimens were incorporated into this NMA. With respect to the overall patient cohort, osimertinib + chemotherapy (CT) was associated with the greatest benefit to progression-free survival (PFS), whereas amivantamab + lazertinib yielded the greatest benefit to overall survival (OS). Osimertinib + CT yielded the best PFS outcomes irrespective of patient gender or EGFR mutation subtype. The combinations of amivantamab + lazertinib and icotinib + CT provided the best respective PFS outcomes in Asian and elderly patient cohorts. With respect to OS outcomes, afatinib + cetuximab provided the best outcomes for 19del and male cases, whereas dacomitinib provided the best OS for females and cases with L858R mutations. The respective gefitinib + CT and erlotinib + bevacizumab regimens were also associated with the greatest improvements in the OS of Asian and elderly cases.

**Conclusions:**

This NMA revealed that cases with EGFR-mutated NSCLC may benefit from different first-line treatment regimens according to their clinicopathological characteristics. On the whole, osimertinib plus CT and amivantamab plus lazertinib emerged as the most noticeable treatment modalities for such cases. (PROSPERO ID: CRD42024506995)

**Supplementary Information:**

The online version contains supplementary material available at 10.1186/s12885-025-15236-z.

## Introduction

Lung cancer bears the unfortunate distinction of being the foremost contributor to cancer-related mortality on a global scale, with NSCLC representing a staggering 85% of diagnosed lung tumor cases [1, 2]. Within the landscape of NSCLC tumors, EGFR mutations emerge as prominent drivers, with the exon 21 Leu858Arg (21L858R) mutation and the exon 19 deletion (19del) standing out as the two most prevalent and clinically significant subtypes [3–5].

EGFR-mutated NSCLC first-line intervention usually includes a variety of EGFR-TKI, namely, 1st-generation EGFR-TKI (gefitinib, erlotinib and icotinib), 2nd-generation EGFR-TKI (dacomitinib and afatinib), and 3rd-generation EGFR-TKI (osimertinib, furmonertinib, almonertinib) [6–9]. Improvement of the efficacy of TKI treatment or to slowing down the onset of therapeutic resistance emerged notable objectives, and researchers have increasingly explored the administration of first-line combination therapies for advanced EGFR-mutated NSCLC, including combining TKIs and chemotherapeutic or anti-angiogenic drugs [10]. In one prior network meta-analysis (NMA) evaluating these various first-line approaches to the therapy of NSCLC cases with advanced EGFR-mutated disease, the investigators identified that osimertinib in isolation exhibited the most favorable patient progression-free survival (PFS), while the combination of gefitinib and pemetrexed-based chemotherapy was linked to superior overall survival (OS) rates [11]. Recently, there have been multiple promising breakthroughs in the first-line therapy of advanced EGFR-mutated NSCLC cases including those derived from the FLAURA2 study [12], MARIPOSA study [13], LASER301 study [14], and IBIO-103 study [15]. This raises the question of whether these results may alter the conclusions of the previous NMA, offering new guidance for therapeutic decision-making. As such, additional comprehensive comparative analyses are essential to delineate the most optimal first-line therapeutic approaches for NSCLC cases carrying EGFR mutations.

At present, first-line EGFR-TKI treatment is recommended for NSCLC cases with both EGFR 19del and EGFR 21L858R mutations, which are classified as completely distinct disease subtypes. Relative to 19del NSCLC, 21L858R disease is associated with more aggressive growth, higher rates of concomitant mutations, reduced EGFR-TKI sensitivity, and other distinct molecular characteristics [16–19]. EGFR mutation subtypes should thus be taken into consideration when selecting patient treatments. There are also differences in the prevalence of driver gene mutations when comparing Asian and non-Asian patient cohorts, and the ARCHER 1050, LUX-Lung3, and FLAURA trials all reported significant differences in therapeutic outcomes when comparing patients of different ethnicities [20–22]. Several other clinicopathological factors beyond ethnicity and EGFR mutation status have also been shown to be relevant to patient prognosis and treatment outcomes, including sex and age, emphasizing their relevance to treatment protocol planning [23, 24]. At present, however, given the ambiguity surrounding the most efficacious treatment strategies for advanced NSCLC cases with distinct EGFR mutation subtypes and clinicopathological profiles, uncertainties persist.

To address these questions, we conducted this NMA, which has been widely used in the absence of head-to-head trial data, to provide an in-depth exploration of the optimal first-line treatment for patients with advanced EGFR-mutant NSCLC. By comparing the efficacy of individual treatment regimens in subgroups of patients with specific clinical and pathological characteristics, this study can provide a solid basis for selecting appropriate treatment regimens for patients.

We present this article in accordance with the PRISMA reporting checklist.

### Methods

Conducted on the basis of the guidelines outlined in the PRISMA (Preferred Reporting Items for Systematic Reviews and Meta-analyses) checklist extension for NMAs, it was attempted to register this study on PROSPERO (ID# CRD42024506995) (Table S1).

### Data sources

The Embase, Cochrane Library, Web of Science, and PubMed databases, in conjunction with conference abstracts, underwent a precise systematic search. This search aimed to involve randomized controlled trials (RCTs) published in English up to December 2023, specifically concentrating on trials that included EGFR-TKI regimens in at least one arm. Full details regarding the utilized search strategy are demonstrated in Table S2. Moreover, the title, abstract, and full text of retrieved articles were independently evaluated by two scholars for the purpose of indicating whether the studies met the defined eligibility criteria, with all discrepancies being resolved by a third investigator.

### Study inclusion

For eligibility in this NMA, studies were required to meet specific criteria: (1) They had to be Phase II or III RCTs concentrating on cases diagnosed with histologically or cytologically confirmed EGFR mutation-positive advanced NSCLC at stage III, IV, or relapsed stage (patients with advanced NSCLC who have relapsed after previous radical treatment); (2) RCTs comparing a minimum of two first-line therapies, at least one of which included an EGFR-TKI; and (3) RCTs that reported at least one outcome, including OS or PFS. Studies were excluded if they were: (1) trials in which the test treatments were provided as maintenance, adjuvant, or neoadjuvant therapy; (2) trials utilizing radiotherapy or immunotherapy; or (3) reviews, editorials, letters, case reports, author responses, or articles not published in English. When more than one study originated from a single trial, only the most complete was included in this NMA.

### Study outcomes and data collection

Data extraction involved the utilization of standardized tables to capture key information, involving study ID, therapeutic modality, first author, publication year, study arms, study design, and total participant count. This extraction process was conducted independently by two investigators. The outcomes for this NMA were patient OS and PFS.

### Quality assessment

It was attempted to figure out the level of the RCTs through the Cochrane Risk of Bias tool according to seven possible sources of bias (random sequence generation, allocation concealment, personnel and participant blinding, selective reporting, incomplete outcome data, outcome assessment blinding, and others). Two investigators independently assigned a high (red), medium (yellow), or low (green) risk of bias to each of these items.

### Data synthesis and assessment

Utilizing STATA 16.0, direct and indirect comparisons of different therapeutic strategies were undertaken through network geometry maps. Survival outcomes were evaluated based on hazard ratios (HRs) for PFS and OS with 95% CIs Evaluating heterogeneity was executed through I^2^ statistic, with I^2^ < 25%, 25–50%, and >50% respectively indicating low, intermediate, and high heterogeneity levels. Random-effect models were used whenever I^2^ >50% [25]. The statistical global inconsistency test was utilized to evaluate the overall network model for global inconsistency, while differences among the direct and indirect comparisons within a closed treatment loop were evaluated through a node-splitting analysis. A P threshold below 0.05 was suggestive of statistical significance. The Markov chain Monte Carlo method was implemented using the “JAGS” (v 4.3.0) and “getmtc” (v 0.8.2) packages in R (v 4.0.2) for fixed effects and consistency models. The establishment of posterior distributions involved the deployment of four separate and independent Markov chains, all concurrently assessing individual outcomes. This rigorous process consisted of 5,000 burn-in iterations followed by 20,000 inference iterations per chain. It was attempted to figure out convergence of the model through trace plots and the Brooks-Gelman-Rubin method. Subsequently, the Bayesian approach was employed to thoroughly analyze the surface under the cumulative ranking curve (SUCRA) for each treatment modality. Higher SUCRA values were interpreted as denoting superior efficacy [26]. Publication bias was examined utilizing funnel plots, and absence of publication bias was presented by symmetrical plots [27].

## Results

**Fig. 1:**
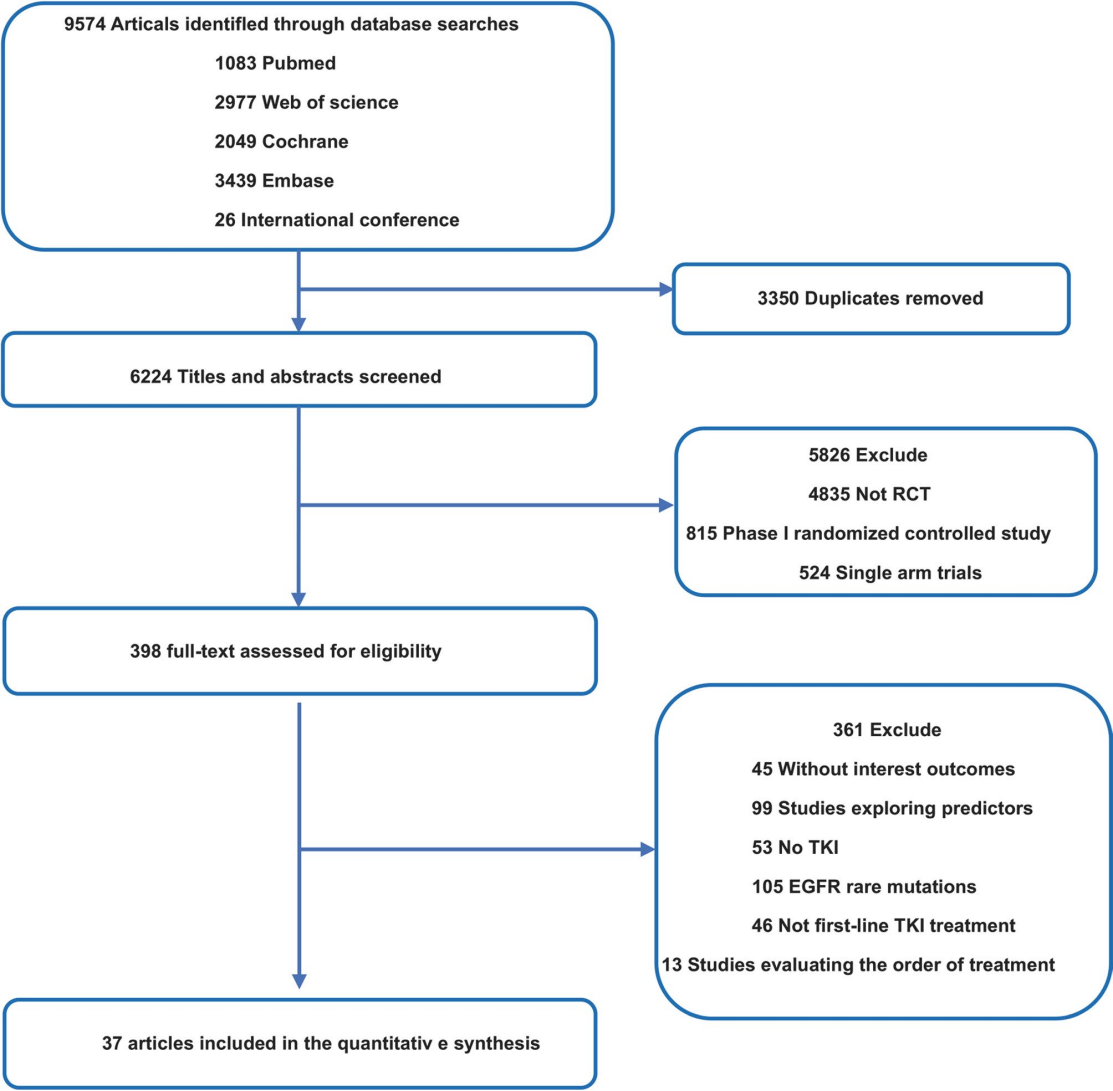
Flow diagram for the selection of eligible studies

**Fig. 2:**
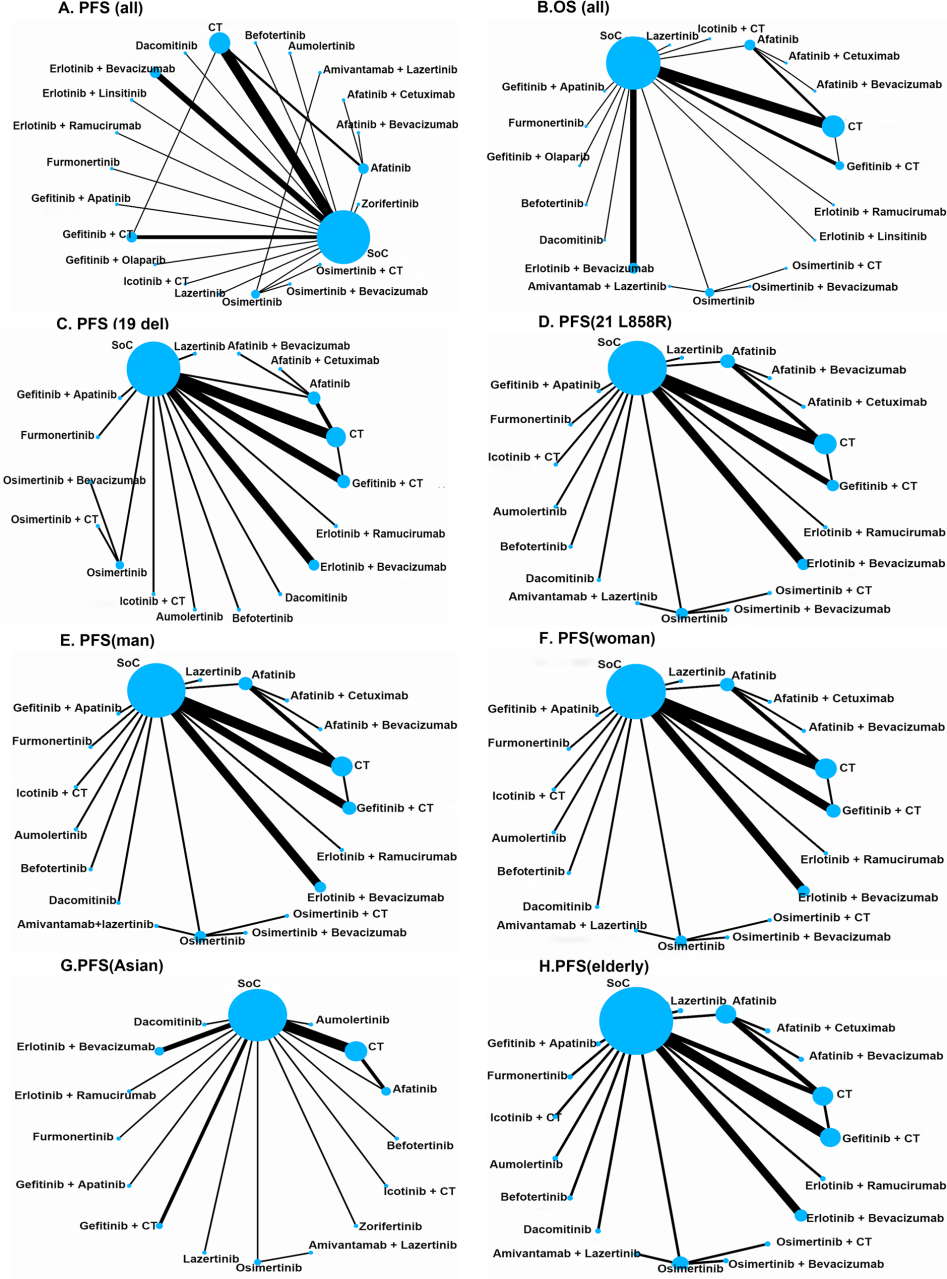
Network map comparing different treatment outcomes in different groups of patients with advanced epidermal growth factor receptor (EGFR) mutated non-small cell lung cancer (NSCLC) Each circular node represents a type of treatment. The node size is proportional to the total number of patients administering a treatment. Each line represents a type of head-to-head comparison. The width of lines is proportional to the total number of studies comparing the connected treatments. **A**. PFS of all patients, **B**. OS of all patients, **C**. PFS of EGFR 19del, **D**. PFS of EGFR L858R, **E**. PFS of man, **F**. PFS of woman, **G**. PFS of Asian, **H**. PFS of elderly. CT, Chemotherapy; SoC, standard of care

In total, this study incorporated 37 RCTs (11 phase II RCTs, 26 phase III RCTs) enrolling 11,539 patients and employing 24 treatment regimens (Table 1) [12–15, 28–60]. A detailed study selection flowchart is presented in Fig. 1. A comparison of the outcomes in this study, which included the PFS and OS of the overall patient cohort and the PFS of male, female, elderly (≥ 65 years), Asia, EGFR 19del, and EGFR L858R cases is presented in Fig. 2. A comparison of the OS of male, female, elderly, Asian, EGFR 19del, and EGFR L858R cases is presented in Figure S1.

**Table 1:** Baseline characteristics of 37 studies

Study ID	Year	First author	Trial phase	Total number	Arm	Neoadjuvant treatment
FLAURA	2018	Jean-Charles Soria	III	279	1	Oral osimertinib
				277	2	Standard oral EGFR-TKI (gefitinib or erlotinib)
CTONG-1509	2018	Qing Zhou	III	157	1	Erlotinib plus bevacizumab
				154	2	Erlotinib
NEJ009	2019	Yukio Hosomi	III	172	1	Gefitinib plus carboplatin and pemetrexed
				170	2	Gefitinib
ENSURE	2015	Yilong Wu	III	110	1	Erlotinib
				107	2	Gemcitabine/cisplatin (GP)
Noronha et al.	2020	Vanita Noronha	III	176	1	Gefitinib plus carboplatin and pemetrexed
				174	2	Gefitinib
Han et al.	2017	Baohui Han	II	40	1	Gefitinib plus carboplatin and pemetrexed
				40	2	Carboplatin and pemetrexed
				41	3	Gefitinib
ARCHER1050	2017	Yi-Long Wu	III	227	1	Dacomitinib
				225	2	Gefitinib
LUX-Lung 3	2016	Martin Schuler	III	230	1	Afatinib
				115	2	Cisplatin and pemetrexed
LUX-Lung 6	2014	Yi-Long Wu	III	242	1	Afatinib
				122	2	Cisplatin plus gemcitabine
LUX-Lung 7	2016	Keunchil Park	IIB	160	1	Afatinib
				159	2	Gefitinib
JO25567	2014	Takashi Seto	II	77	1	Erlotinib plus bevacizumab
				77	2	Erlotinib
NEJ026	2019	Haruhiro Saito	III	112	1	Erlotinib plus bevacizumab
				112	2	Erlotinib
RELAY	2019	Kazuhiko Nakagawa	III	224	1	Erlotinib plus ramucirumab
				225	2	Erlotinib
ACTIVE	2021	Hongyun Zhao	III	157	1	Gefitinib and apatinib
				156	2	Gefitinib
WJOG9717L	2022	Hirotsugu Kenmotsu	II	61	1	Osimertinib plus bevacizumab
				61	2	Osimertinib
AENEAS	2022	Shun Lu	III	214	1	Aumolertinib
				215	2	Gefitinib
BEVERLY	2022	Maria Carmela Piccirillo	III	80	1	Erlotinib plus bevacizumab
				80	2	Erlotinib
AfaBev-CS	2023	Takashi Ninomiya	II	49	1	Afatinib plus bevacizumab
				50	2	Afatinib
FLAURA2	2023	David Planchard	III	279	1	Osimertinib plus chemotherapy
				278	2	Osimertinib
MARIPOSA	2023	Byoung Chul Cho	III	429	1	Amivantamab plus lazertinib
				429	2	Osimertinib
FURLONG	2022	Yuankai Shi	III	178	1	Furmonertinib
				179	2	Gefitinib
Cheng et al.	2016	Ying Cheng	II	129	1	Gefitinib plus pemetrexed
				66	2	Gefitinib
LASER301	2023	Byoung Chul Cho	III	196	1	Lazertinib
				197	2	Gefitinib
Wu et al.	2023	Yi-Long Wu	III	220	1	Zorifertinib
				219	2	Gefitinib or erlotinib
IBIO-103	2023	Shun Lu	III	182	1	Befotertinib
				180	2	Icotinib
CONVINCE	2017	Y K Shi	III	148	1	Icotinib
				137	2	Cisplatin/pemetrexed
EURTAC	2012	Rafael Rosell	III	86	1	Erlotinib
				87	2	Standard chemotherapy
OPTIMAL	2011	Caicun Zhou	III	82	1	Erlotinib
				72	2	Standard chemotherapy
NEJ002	2010	A. Inoue	III	114	1	Gefitinib
				114	2	Standard chemotherapy
WJTOG3405	2010	Tetsuya Mitsudomi	III	86	1	Gefitinib
				86	2	Cisplatin plus docetaxel
IPASS	2009	Tony S. Mok	III	609	1	Gefitinib
				608	2	Carboplatin and paclitaxel
Xu et al.	2019	Lisheng Xu	II	90	1	Icotinib plus pemetrexed and carboplatin
				89	2	Icotinib
Stinchcombe et al.	2019	Thomas E Stinchcombe	II	43	1	Erlotinib plus bevacizumab
				45	2	Erlotinib
SWOGS1403	2020	Sarah B. Goldberg	II	83	1	Afatinib plus cetuximab
				85	2	Afatinib
Leighl et al.	2017	Natasha B. Leighl	II	44	1	Erlotinib plus linsitinib
				44	2	Erlotinib
GOAL	2020	Rosario Garcia-Campelo	II	91	1	Gefitinib and olaparib
				91	2	Gefitinib
Cortot et al.	2021	Alexis B Cortot	II	59	1	Afatinib plus cetuximab
				58	2	Afatinib
AGAIN	2023	Shintaro Kanda	III	251	1	Gefitinib/Osimertinib + Cisplatin and pemetrexed
				251	2	Gefitinib/Osimertinib

### Evaluating heterogeneity/inconsistency and risk of bias

**Fig. 3:**
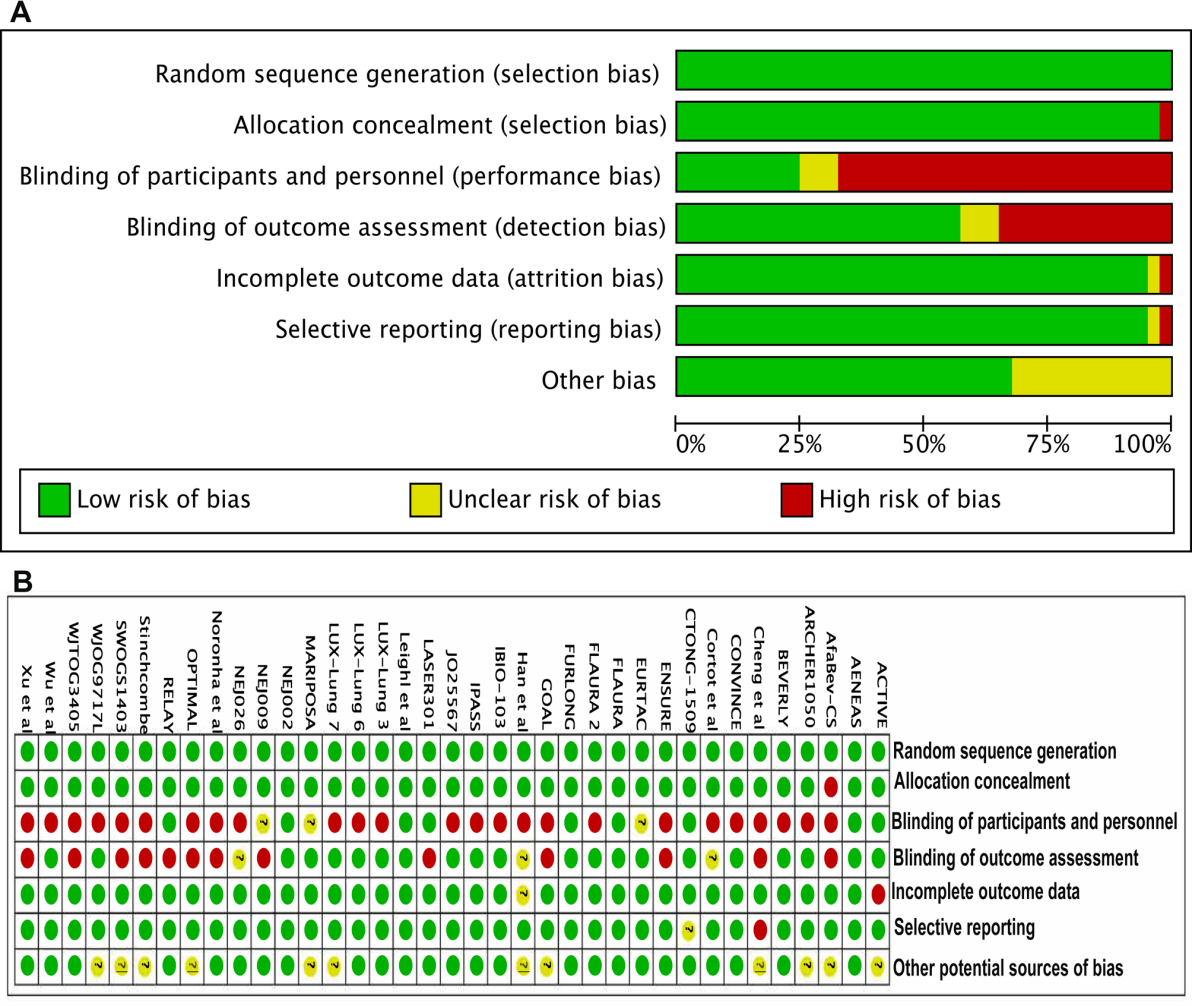
Summary of risk-of-bias analysis. **A**. Risk of bias graph, **B**. Risk of bias summary. Green represents a low risk of bias, yellow an unclear risk, and red a high

Risk of bias for the included RCTs exhibited that 4 presented with a high risk of bias, while 3 had a risk that was unclear, and the other exhibited a low risk of bias (Fig. 3).

Heterogeneity analyses exhibited low to moderate heterogeneity in most cases, although high heterogeneity was exhibited in few instances, and a random-effects model was thus utilized for the purpose of conducting these analyses (Figure S2-S7).

The absence of significant inconsistency was noteworthy on the basis of outcomes of global inconsistency test (*P* > 0.05) (Figure S8, Figure S9). As such, these analyses were performed using a consistency model.

### NMA of PFS and OS in all cases

**Fig. 4:**
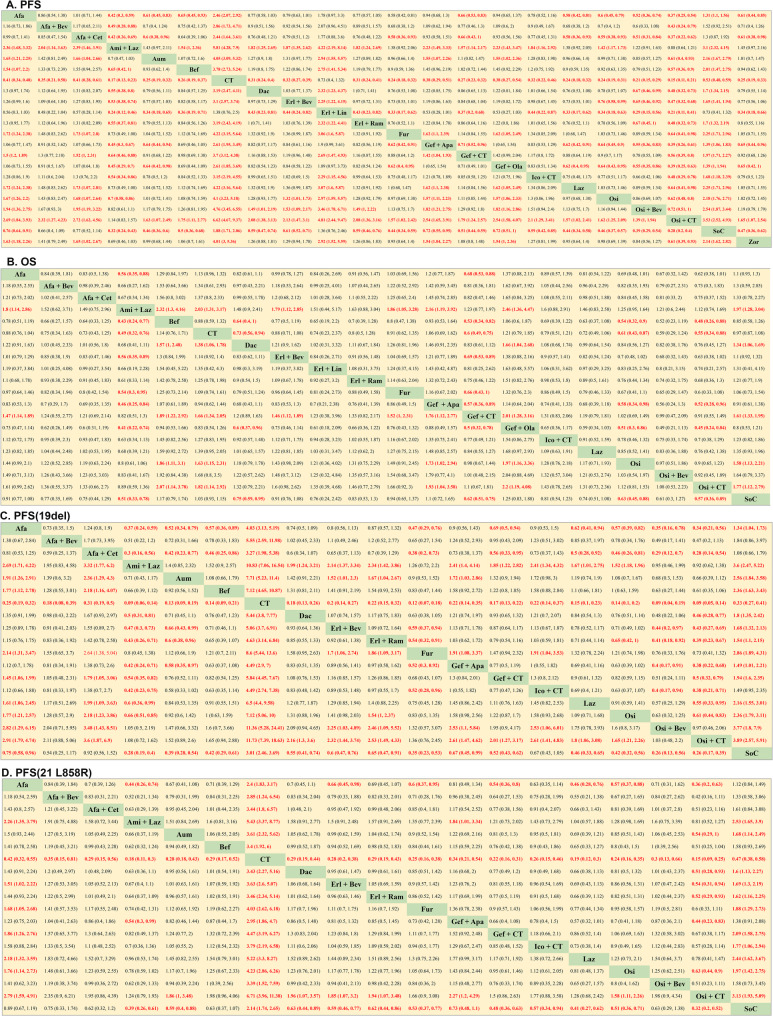
Pooled estimates of the network meta-analysis were based on the general population and different EGFR mutation subtypes **A**. Pooled hazard ratios (95% credible intervals(CI)) for PFS in all patients; **B**. Pooled hazard ratios (95% CI) for OS in all patients; **C**. Pooled hazard ratios (95% CI) for PFS in patients with EGFR 19del; **D**. Pooled hazard ratios (95% CI) for PFS in patients with EGFR 21 L858R. Afa, Afatinib; Afa + Bev, Afatinib + bevacizumab; Afa + Cet, Afatinib + cetuximab; Ami + Laz, Amivantamab + lazertinib; Aum, Aumolertinib; Bef, Befotertinib; CT, Chemotherapy; Dac, Dacomitinib; Erl + Bev, Erlotinib + bevacizumab; Erl + Lin, Erlotinib + linsitinib; Erl + Ram, Erlotinib + ramucirumab; Fum, Furmonertinib; Gef + Apa, Gefitinib + apatinib; Gef + CT, Gefitinib + chemotherapy; Gef + Ola Gefitinib + olaparib; Ico + CT, Icotinib + chemotherapy; Laz, Lazertinib; Osi, Osimertinib; Osi + Bev, Osimertinib + bevacizumab; Osi + CT, Osimertinib + chemotherapy; SoC, Standard of care (gefitinib, erlotinib, icotinib); Zor, Zorifertinib

Relative to osimertinib (HR, 0.70, 95% CI, 0.58–0.86) and befotertinib (HR, 0.65, 95% CI, 0.42–1.00.42.00), amivantamab + lazertinib was associated with improved PFS. EGFR-mutated advanced NSCLC cases treated with osimertinib + CT exhibited PFS significantly better than that of cases treated with aumolertinib (HR, 0.61, 95% CI, 0.40–0.93), furmonertinib (HR, 0.64, 95% CI, 0.41–0.98), lazertinib (HR, 0.64, 95% CI, 0.41–0.98), osimertinib (HR, 0.62, 95% CI, 0.48–0.80), osimertinib + bevacizumab (HR, 0.72, 95% CI, 0.51–1.00.51.00), befotertinib (HR, 0.57, 95% CI, 0.36–0.90), and zorifertinib (HR, 0.61, 95% CI, 0.39–0.93). The absence of differences particularly in PFS was noteworthy when comparing osimertinib, aumolertinib, furmonertinib, befotertinib, lazertinib, zorifertinib, and osimertinib + bevacizumab (Fig. 4 A).

Osimertinib (HR, 0.54, 95% CI, 0.32–0.90) and osimertinib + CT (HR, 0.48, 95% CI, 0.26–0.88) exhibited correlation with significant improvements in the OS of advanced EGFR-mutated NSCLC cases relative to befotertinib. Amivantamab + lazertinib was linked to significantly greater OS relative to furmonertinib (HR, 0.54, 95% CI, 0.30–0.95) and befotertinib (HR, 0.43, 95% CI, 0.24–0.77). The absence of differences particularly in OS was noteworthy when comparing the osimertinib, osimertinib + bevacizumab, osimertinib + CT, lazertinib, and amivantamab + lazertinib regmines (Fig. 4B).

### NMA of PFS of EGFR mutation subtype

Amivantamab + lazertinib significantly outperformed both lazertinib (HR, 0.60, 95% CI, 0.36–0.99) and osimertinib (HR, 0.66, 95% CI, 0.51–0.89) with respect to its ability to prolong NSCLC cases’ PFS with EGFR 19del mutations. An extension of PFS was recorded in the osimertinib + CT group relative to the lazertinib (HR, 0.55, 95% CI, 0.33–0.95), osimertinib (HR, 0.61, 95% CI, 0.44–0.83), gefitinib + CT (HR, 0.50, 95% CI, 0.32–0.79), icotinib + CT(HR, 0.38, 95% CI, 0.21–0.71), and erlotinib + bevacizumab (HR, 0.43, 95% CI, 0.27–0.69) groups. However, the absence of differences particularly in PFS was noteworthy among EGFR 19del cases when comparing the aumolertinib, furmonertinib, befotertinib, lazertinib, osimertinib, and osimertinib + bevacizumab regimens (Fig. 4 C).

Osimertinib + CT provided EGFR L858R NSCLC cases with significantly extended PFS relative to osimertinib (HR, 0.63, 95% CI, 0.44–0.90), aumolertinib (HR, 0.54, 95% CI, 0.29–1.00.29.00), dacomitinib (HR, 0.51, 95% CI, 0.28–0.93), erlotinib + bevacizumab (HR, 0.54, 95% CI, 0.31–0.94), erlotinib + ramucirumab (HR, 0.52, 95% CI, 0.29–0.93), and gefitinib + apatinib (HR, 0.44, 95% CI, 0.23–0.83). The absence of differences particularly in PFS was noteworthy when comparing the aumolertinib, befotertinib, dacomitinib, furmonertinib, lazertinib, osimertinib, osimertinib + bevacizumab, amivantamab + lazertinib, erlotinib + bevacizumab, gefitinib + CT, and icotinib + CT regimens (Fig. 4D).

### NMA of PFS in different gender subgroups

**Fig. 5:**
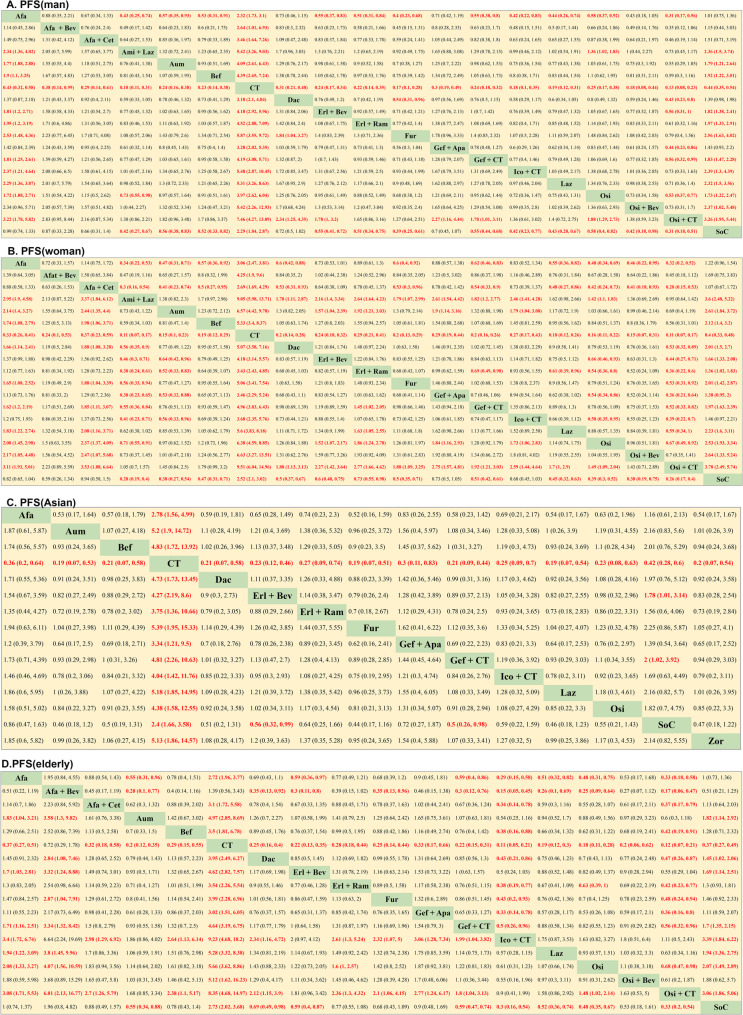
Pooled estimates for the network meta-analysis were based on gender, race, and age **A**. Pooled hazard ratios (95% CI) for PFS in man patients; **B**. Pooled hazard ratios (95% CI) for PFS in woman patients; **C**. Pooled odds ratios (95% CI) for Asian patients; **D**. Pooled odds ratios (95% CI) for elderly patients

Among males, furmonertinib (HR, 0.54, 95% CI, 0.31–0.96) and osimertinib + CT (HR, 0.43, 95% CI, 0.23–0.80) exhibited correlation with significantly extended PFS relative to dacomitinib. The amivantamab + lazertinib (HR, 0.73, 95% CI, 0.55–0.98) and osimertinib + CT (HR, 0.53, 95% CI, 0.37–0.77) groups exhibited PFS significantly longer than that for the osimertinib group. Significantly better PFS was also observed in the osimertinib + CT group relative to the erlotinib + bevacizumab (HR, 0.56, 95% CI, 0.31–1.00.31.00), gefitinib + apatinib (HR, 0.44, 95% CI, 0.23–0.86), and gefitinib + CT (HR, 0.56, 95% CI, 0.32–0.99) groups. The absence of differences particularly in PFS extension yielded by the aumolertinib, befotertinib, furmonertinib, lazertinib, osimertinib, and osimertinib + bevacizumab regimens was noteworthy for male cases, but there was a trend toward superiority of furmonertinib over the other interventions (Fig. 5 A).

Amivantamab + lazertinib significantly outperformed dacomitinib (HR, 0.56, 95% CI, 0.35–0.99), erlotinib + bevacizumab (HR, 0.43, 95% CI, 0.30–0.71), furmonertinib (HR, 0.56, 95% CI, 0.33–0.94), gefitinib + CT(HR, 0.55, 95% CI, 0.36–0.84), icotinib + CT (HR, 0.41, 95% CI, 0.23–0.71), and osimertinib (HR, 0.71, 95% CI, 0.55–0.91) with respect to the PFS outcomes for female cases. Significantly better PFS was observed in both the aumolertinib and osimertinib groups relative to the erlotinib + bevacizumab (aum: HR, 0.64, 95% CI, 0.42–0.96; osi: HR, 0.66, 95% CI, 0.46–0.93) and icotinib + CT (aum: HR, 0.56, 95% CI, 0.33–0.96; osi: HR, 0.58, 95% CI, 0.35–0.95) groups. Osimertinib + CT treatment yielded significantly better improvements in patient PFS relative to treatment with dacomitinib (HR, 0.53, 95% CI, 0.32–0.89), erlotinib + bevacizumab (HR, 0.44, 95% CI, 0.27–0.71), furmonertinib (HR, 0.53, 95% CI, 0.31–0.92), gefitinib + CT (HR, 0.52, 95% CI, 0.33–0.82), icotinib + CT (HR, 0.39, 95% CI, 0.22–0.70), lazertinib (HR, 0.59, 95% CI, 0.34–1.00.34.00), and osimertinib (HR, 0.67, 95% CI, 0.49–0.92)(Fig. 5B).

### NMA of PFS is Asian and elderly patient subgroups

Treatment with erlotinib + bevacizumab (HR, 0.56, 95% CI, 0.32–0.99) and gefitinib + CT (HR, 0.50, 95% CI, 0.26–0.98) exhibited correlation with significantly better improvements in the PFS of Asian cases relative to SoC. The absence of differences particularly in PFS was noteworthy among the aumolertinib, afatinib, befotertinib, dacomitinib, lazertinib, furmonertinib, osimertinib, and zorifertinib regimens, although furmonertinib tended to outperform other treatments (Fig. 5 C).

Among elderly cases, icotinib + CT treatment was associated with significantly better improvements in patient PFS relative to afatinib (HR, 0.29, 95% CI, 0.15–0.58), afatinib + bevacizumab (HR, 0.15, 95% CI, 0.05–0.45), befotertinib (HR, 0.38, 95% CI, 0.16–0.88), dacomitinib (HR, 0.43, 95% CI, 0.21–0.86), furmonertinib (HR, 0.43, 95% CI, 0.20–0.93), and gefitinib + CT (HR, 0.50, 95% CI, 0.26–0.96). Relative to afatinib (HR, 0.33, 95% CI, 0.18–0.58), afatinib + bevacizumab (HR, 0.17, 95% CI, 0.06–0.47), befotertinib (HR, 0.42, 95% CI, 0.19–0.91), dacomitinib (HR, 0.47, 95% CI, 0.26–0.87), furmonertinib (HR, 0.48, 95% CI, 0.24–0.94), osimertinib (HR, 0.68, 95% CI, 0.47–0.98), and gefitinib + CT(HR, 0.56, 95% CI, 0.32–0.96), osimertinib + CT was associated with significantly better improvements in PFS among elderly cases. Significantly longer PFS was observed in the dacomitinib (HR, 0.35, 95% CI, 0.13–0.92), aumolertinib (HR, 0.28, 95% CI, 0.10–0.77), furmonertinib (HR, 0.35, 95% CI, 0.13–0.96), lazertinib (HR, 0.26, 95% CI, 0.10–0.69), and osimertinib (HR, 0.25, 95% CI, 0.09–0.64) groups relative to the afatinib + bevacizumab group (Fig. 5D).

### NMA of OS of EGFR mutation subtype

The absence of differences particularly in OS extension was noteworthy among EGFR 19del cases with the exception of the significantly improved OS for cases in the afatinib group relative to those in the CT group (Figure S10A).

While there afatinib, afatinib + cetuximab, dacomitinib, erlotinib + bevacizumab, erlotinib + CT, osimertinib, and SoC did not differ significantly in their ability to improve the OS of EGFR L858R cases, dacomitinib tended to outperform other therapeutic modalities (Figure S10B).

### NMA of OS in different gender subgroups

Among male cases, afatinib significantly outperformed CT (HR, 0.73, 95% CI, 0.55–0.97) with respect to its ability to improve OS. Relative to SoC (HR, 0.55, 95% CI, 0.36–0.83) and CT (HR, 0.53, 95% CI, 0.32–0.87), gefitinib + CT yielded significantly better OS (Figure S10C). Among female cases, dacomitinib was associated with significant improvements in OS relative to SoC, and it also tended to outperform other treatments (Figure S10D).

### NMA of OS in Asian and elderly patient subgroups

In the Asian patient population, gefitinib + CT significantly outperformed afatinib (HR, 0.73, 95% CI, 0.53–0.97), befotertinib (HR, 0.58, 95% CI, 0.37–0.90), erlotinib + bevacizumab (HR, 0.74, 95% CI, 0.55–0.99), gefitinib + apatinib (HR, 0.62, 95% CI, 0.39–0.99), SoC (HR, 0.68, 95% CI, 0.55–0.84), and osimertinib (HR, 0.69, 95% CI, 0.49–0.97) as a menas of improving patient OS. The OS of cases in the dacomitinib treatment group was also significantly better than that of cases in the SoC group (HR, 0.76, 95% CI, 0.58–0.99) (Figure S10E).

Erlotinib + bevacizumab was associated with superior OS outcomes relative to CT in elderly cases and tended to outperform other therapeutic regimens (Figure S10F).

### Efficacy rankings

With respect to PFS among the overall patient population, EGFR 19del cases, EGFR L858R cases, and women, osimertinib + CT yielded the best outcomes, followed by amivantamab + lazertinib. In the Asian population, amivantamab + lazertinib yielded the best outcomes, followed by osimertinib + CT. Among elderly cases, icotinib + CT yielded the best PFS outcomes, followed by osimertinib + CT (Figure S11).

With respect to OS among the overall patient cohort, amivantamab + lazertinib yielded the best outcomes, followed by osimertinib + CT treatment. Among EGFR 19del and male cases, the afatinib + cetuximab regimen yielded the best OS, while dacomitinib was associated with the best outcomes among EGFR L858R and female cases. Among Asian and elderly cases, gefitinib + CT and erlotinib + bevacizumab, respectively, exhibited correlation with the greatest OS outcomes (Figure S12).

### Convergence and publication bias analyses

Good convergence was evident for all comparisons in the present NMA based on the established Brooks-Gelman-Rubin diagnostic plots (Figure S13, Figure S14) and trace plots (Figure S15-S20), and the symmetrical funnel plots were suggestive of no publication bias (Figure S21, Figure S22).

## Discussion

Multiple EGFR-TKI therapeutic regimens have been designed to date and demonstrated to afford survival benefits to NSCLC cases whose tumors harbor EGFR mutations. Direct comparisons of these extant therapeutic modalities, however, are largely lacking, presenting clinicians with an immense challenge when seeking to select the best first-line regimen for use when treating a given patient. To address this issue, it was attempted to conduct the present investigation for comparable analysis of the efficacy of 24 different treatment strategies through an NMA approach, revealing that osimertinib + CT afforded the greatest benefits to the PFS of treated cases, although amivantamab + lazertinib may be associated with greater improvements in OS.

Within the encompassing cohort of EGFR-mutated NSCLC cases examined, the treatment regimens involving osimertinib + CT and amivantamab + lazertinib emerged as particularly beneficial, showcasing notable enhancements in both PFS and OS. Notably, the FLAURA study underscored the significant extension of OS and PFS in EGFR-mutated NSCLC cases with first-line osimertinib treatment [28]. This efficacy profile has led to the designation of osimertinib as the preferred therapeutic choice for cases with EGFR mutations according to the NCCN guidelines. Despite this, the persistent challenge of acquired resistance to EGFR-TKIs persists as a significant clinical concern. In response, there is ongoing exploration into combinatorial approaches involving these TKIs with other modalities to counteract this resistance phenomenon. Building on these findings, the FLAURA 2 study demonstrated that combining osimertinib and CT led to even greater improvements in patient outcomes relative to osimertinib alone, prolonging median patient PFS by approximately 9 months while lowering the risk of death or disease progression by 38% [12]. Amivantamab is a bispecific monoclonal antibody that binds to both EGFR and the c-MET receptor proteins on the cell surface, thereby blocking downstream signal transmission and inhibiting the proliferation of tumor cells expressing these proteins. The potent third-generation nervous system osmotic TKI lazertinib is capable of targeting EGFR-sensitive mutations and T790M mutations without impacting the activity of wild-type EGFR. In the phase III MARIPOSA trial, the amalgamation of amivantamab and lazertinib showcased a median PFS of 23.7 months, in stark contrast to the 16.6 months noted with osimertinib monotherapy [13]. Osimertinib and bevacizumab interact with a shared cascade of signaling pathways downstream from their targets. This synergy enables their combined application to effectively disrupt EGFR/VEGF signaling activity, potentially forestalling the onset of drug resistance and yielding superior antitumor efficacy [61, 62]. The latest results of a comprehensive NMA analysis have shown that osimertinib in combination with bevacizumab may be superior to other treatments regarding extension of PFS for cases with EGFR-mutated advanced NSCLC. In the present study, however, osimertinib + CT and amivantamab + lazertinib yielded greater improvements in the PFS an OS of cases relative to the combination of osimertinib + bevacizumab. These high-level findings, however, warrant more granular examination. If amivantamab + lazertinib is applied as a first-line treatment, for instance, the utilization of the MARIPOSA-2 protocols (amivantamab + CT ± lazertinib) would be inappropriate following patient relapse. As an alternative, the approved osimertinib + CT may be selected as the first-line approach, with combination amivantamab treatment being explored as a second-line option.

Several trials to date have shown that EGFR-TKI efficacy varies as a function of the subtype of EGFR mutation present in the target tumor, with cases harboring 19del mutations attaining greater benefits from TKI treatment relative to 21L858R cases [63–65]. While some past studies have compared the most effective first-line treatments for advanced NSCLC cases with these two types of EGFR driver mutations [66, 67], the emergence of more recent data from several major RCTs has largely rendered these earlier findings obsolete. The present NMA indicated that osimertinib + CT and amivantamab + lazertinib are still the best first-line option to improve the PFS of cases harboring either EGFR 19del or L858R mutations, in line with what was observed in the overall study population. Moreover, single-agent third-generation TKI treatment was associated with a promising PFS benefit among 19del cases, whereas combination therapy yielded better survival outcomes for L858R cases. This may be related to the more aggressive growth of 21L858R tumors, which also exhibit higher co-mutation rates and worse TKI affinity [68–70]. Notably, however, 19del cases were found to benefit to a greater extent from osimertinib + bevacizumab treatment relative to the 21 L858R cases. This raises the possibility that combination chemotherapy is superior to combined anti-angiogenic treatment as a TKI combination strategy for L858R cases. The mechanistic basis for this finding, however, remains uncertain, underscoring an important avenue for further research.

A growing wealth of evidence suggests that particular clinicopathological characteristics including sex, age, and ethnicity can all shape treatment outcomes and patient prognosis such that these factors should be considered when selecting the best therapeutic modalities for these individuals. The results of this NMA revealed that osimertinib + CT yielded the greatest improvements in PFS among both male and female cases. However, males attained greater benefits from furmonertinib relative to female cases. TKI combination therapies also tended to be the most highly ranked regimens for male cases, whereas single-agent TKI treatments tended to provide better outcomes for female cases. This may suggest that female cases exhibit greater TKI sensitivity relative to male cases, in line with past reports [71, 72]. Among Asian cases, the present analyses revealed that the two regimens associated with the greatest PFS benefits were the same as those for the overall patient population. However, Asian cases attained lower levels of benefit from osimertinib monotherapy relative to the overall study cohort. Furmonertinib and aumolertinib may therefore be better options for the single-agent TKI management of Asian cases. Icotinib + CT and osimertinib + CT yielded the greatest PFS benefits to elderly cases in this study, partially echoing past reports [67]. Osimertinib was also identified as the best alternative option for the treatment of elderly cases unable to tolerate combination therapy.

The limitations of the current investigation should be pointed out. Notably, the categorization of gefitinib, erlotinib, and icotinib into a single treatment group may introduce a potential bias. Nonetheless, it is essential to highlight that multiple studies have consistently shown similar safety and efficacy profiles across these first-generation TKIs [46, 66, 73, 74]. Secondly, this NMA was based only on available clinical trial data, but it is nonetheless susceptible to the effects of confounding factors. Third, this analysis could not analyze adverse effects in subgroups of cases according to particular clinicopathological characteristics, as these events primarily occurred in the general population rather than in patient subgroups. Fourth, while OS is regarded as the gold standard metric by which to analyze antitumor therapies, the OS data for some of the included clinical trials remains relatively immature such that this may have introduced some degree of heterogeneity into the results. As such, PFS was the primary outcome indicator evaluated in this study, underscoring an important avenue for future research. Fifth, this network meta-analysis has inherent limitations. The trials we included were mainly conducted at different times, and their molecular diagnostic methods were mostly PCR rather than the more comprehensive NGS. Therefore, we are unable to obtain detailed patient-level data on specific EGFR mutation subtypes (for example, uncommon exon 19 deletion mutations [75] that may vary in response to treatment) or molecular co-mutations (such as MET, TP53, etc.). The heterogeneity of these biological factors may significantly affect the treatment outcome [76, 77]. For instance, for the Amivantamab + Lazertinib combination regimen, existing evidence suggests that the patient population with high MET protein expression (IHC positive) may benefit more significantly [78], but our analysis cannot be conducted at this subdivision level. Future network meta-analyses of individual patient data or aggregated data studies targeting specific biomarker subgroups are crucial for revealing the relative efficacy of different treatment regimens in subgroups with more precisely defined molecules.

## Conclusions

This NMA demonstrates that the optimal first-line treatment for advanced EGFR-mutated NSCLC may vary based on specific clinicopathological characteristics. The key findings can be summarized as follows:

- Broadly superior regimens: Osimertinib plus CT and amivantamab plus lazertinib emerged as the most notable regimens, demonstrating superior PFS and OS benefits, respectively, in the overall population.
- PFS benefits: Osimertinib plus CT consistently showed the greatest PFS benefit across different genders and EGFR mutation subtypes.
- OS benefits based on subgroups: The greatest OS benefits were associated with:
  - 19del mutations and male patients: Afatinib + cetuximab.
  - L858R mutations and female patients: Dacomitinib.
  - Asian patients: Gefitinib + CT.
  - Elderly patients: Erlotinib + bevacizumab.

These findings underscore the potential for personalized first-line treatment strategies.

## Supplementary Information

Supplementary Material 1: Table S1. PRISMA extension checklist for this network meta-analysis. Table S2. Search strategy. Figure S1. Network map comparing different treatment outcomes in different groups. Figure S2-S7. Heterogeneity analysis. Figure S8, S9. Inconsistency analysis. Figure S10. Pooled estimates of the network meta-analysis. Figure S11, S12. Ranking diagram for network meta-analysis. Figure S13, S14. Brooks-Gelman-Rubin diagnostic plots for evaluating model convergence. Figure S15-S20. Trace plot for evaluating model convergence. Figure S21, S22. Funnel plot for evaluating publication bias.

Supplementary Material 2.

Supplementary Material 3.

Supplementary Material 4.

Supplementary Material 5.

Supplementary Material 6.

Supplementary Material 7.

Supplementary Material 8.

## Data Availability

The datasets used and/or analysed during the current study are available from the corresponding author on reasonable request.
