# Supplementary material for: Assessing first-line treatment for advanced EGFR-mutated NSCLC in diverse clinicopathological subgroups: a systematic review and network meta-analysis
Source: BMC Cancer. 2025 Nov 14;25:1767. doi: 10.1186/s12885-025-15236-z (PMC12619336; doi:10.1186/s12885-025-15236-z)

Title: Assessing first-line treatment for advanced EGFR-mutated NSCLC in diverse clinicopathological subgroups: a systematic review and network meta-analysis
Running title: Comparison of treatment of EGFR-mutated NSCLC

Ting Mei[1,2], MD, Ting Wang[1,2], MD, Qinghua Zhou[1,2]*, MD,PhD

1. Lung Cancer Center/Lung Cancer Institute, West China Hospital, Sichuan University
2. Department of Medical Oncology, Cancer Center, West China Hospital, Sichuan University, Chengdu, Sichuan, China

*Corresponding Author: Qinghua Zhou, Lung Cancer Center, West China Hospital, Sichuan University, Chengdu, 610000, China
Email: prof_qh_zhou@126.com
Tel: +86-28-85423581 Fax: +86-28-85423571

Supplementary Online Content

Table S1. PRISMA extension checklist for this network meta-analysis………………………….…….2-6

Table S2 Search strategy…………………………………………………………………………..….7-8

Figure S1 Network map comparing different treatment outcomes in different groups…………… 9

Figure S2-S7 Heterogeneity analysis………………………………………………………………..10-15

Figure S8, S9 Inconsistency analysis…………………………………………………………...…….16-17

Figure S10 Pooled estimates of the network meta-analysis…………………………….……………18

Figure S11, S12 Ranking diagram for network meta-analysis…………………………………..……19-20

Figure S13, S14 Brooks-Gelman-Rubin diagnostic plots for evaluating model convergence………21-22

Figure S15-S19 Trace plot for evaluating model convergence……………………………………23-27

Figure S20, S21 Funnel plot for evaluating publication bias……………………………….....…..28-29

Table S1. Checklist of the PRISMA extention for network meta-analysis

Section/Topic	Item #	Checklist Item	Reported on Page #
**TITLE**			
Title	1	Identify the report as a systematic review *incorporating a network meta-analysis (or related form of meta-analysis).*	**1**
**ABSTRACT**			**2-3**
Structured summary	2	Provide a structured summary including, as applicable:<br>**Background:** main objectives<br>**Methods:** data sources; study eligibility criteria, participants, and interventions; study appraisal; and *synthesis methods, such as network meta-analysis.*<br>**Results:** number of studies and participants identified; summary estimates with corresponding confidence/credible intervals; *treatment rankings may also be discussed. Authors may choose to summarize pairwise comparisons against a chosen treatment included in their analyses for brevity.*<br>**Discussion/Conclusions:** limitations; conclusions and implications of findings.<br>**Other:** primary source of funding; systematic review registration number with registry name.	
**INTRODUCTION**			
Rationale	3	Describe the rationale for the review in the context of what is already known*, including mention of why a network meta-analysis has been conducted.*_	**4-5**
Objectives	4	Provide an explicit statement of questions being addressed, with reference to participants, interventions, comparisons, outcomes, and study design (PICOS).	**5-6**
**METHODS**			
Protocol and registration	5	Indicate whether a review protocol exists and if and where it can be accessed (e.g., Web address); and, if available, provide registration information, including registration number.	**6**

Eligibility criteria	6	Specify study characteristics (e.g., PICOS, length of follow-up) and report characteristics (e.g., years considered, language, publication status) used as criteria for eligibility, giving rationale. *Clearly describe eligible treatments included in the treatment network, and note whether any have been clustered or merged into the same node (with justification).*_	**6-7**
Information sources	7	Describe all information sources (e.g., databases with dates of coverage, contact with study authors to identify additional studies) in the search and date last searched.	**7**
Search	8	Present full electronic search strategy for at least one database, including any limits used, such that it could be repeated.	Table S2
Study selection	9	State the process for selecting studies (i.e., screening, eligibility, included in systematic review, and, if applicable, included in the meta-analysis).	**6-7**
Data collection process	10	Describe method of data extraction from reports (e.g., piloted forms, independently, in duplicate) and any processes for obtaining and confirming data from investigators.	**7**
Data items	11	List and define all variables for which data were sought (e.g., PICOS, funding sources) and any assumptions and simplifications made.	**7**
**Geometry of the network**	**S1**	Describe methods used to explore the geometry of the treatment network under study and potential biases related to it. This should include how the evidence base has been graphically summarized for presentation, and what characteristics were compiled and used to describe the evidence base to readers.	**7-8**
Risk of bias within individual studies	12	Describe methods used for assessing risk of bias of individual studies (including specification of whether this was done at the study or outcome level), and how this information is to be used in any data synthesis.	**7**
Summary measures	13	State the principal summary measures (e.g., risk ratio, difference in means). *Also describe the use of additional summary measures assessed, such as treatment rankings and surface under the cumulative ranking curve (SUCRA) values, as well as modified approaches used to present summary*	**7-8**

		findings from meta-analyses.	
Planned methods of analysis	14	Describe the methods of handling data and combining results of studies for each network meta-analysis. This should include, but not be limited to:<br>• *Handling of multi-arm trials;*<br>• *Selection of variance structure;*<br>• *Selection of prior distributions in Bayesian analyses; and*<br>• *Assessment of model fit.*	**7-8**
**Assessment of Inconsistency**	**S2**	Describe the statistical methods used to evaluate the agreement of direct and indirect evidence in the treatment network(s) studied. Describe efforts taken to address its presence when found.	7-8
Risk of bias across studies	15	Specify any assessment of risk of bias that may affect the cumulative evidence (e.g., publication bias, selective reporting within studies).	**7**
Additional analyses	16	Describe methods of additional analyses if done, indicating which were pre-specified. This may include, but not be limited to, the following:<br>• Sensitivity or subgroup analyses;<br>• Meta-regression analyses;<br>• *Alternative formulations of the treatment network; and*<br>• *Use of alternative prior distributions for Bayesian analyses (if applicable).*_	**10-14**
**RESULTS†**			
Study selection	17	Give numbers of studies screened, assessed for eligibility, and included in the review, with reasons for exclusions at each stage, ideally with a flow diagram.	**9**
**Presentation of network structure**	**S3**	Provide a network graph of the included studies to enable visualization of the geometry of the treatment network.	**9**
**Summary of network geometry**	**S4**	Provide a brief overview of characteristics of the treatment network. This may include commentary on the abundance of trials and randomized patients for the different interventions and pairwise	**9**

		comparisons in the network, gaps of evidence in the treatment network, and potential biases reflected by the network structure.	
Study characteristics	18	For each study, present characteristics for which data were extracted (e.g., study size, PICOS, follow-up period) and provide the citations.	**Table 1**
Risk of bias within studies	19	Present data on risk of bias of each study and, if available, any outcome level assessment.	**9**
Results of individual studies	20	For all outcomes considered (benefits or harms), present, for each study: 1) simple summary data for each intervention group, and 2) effect estimates and confidence intervals. *Modified approaches may be needed to deal with information from larger networks.*	**9-14**
Synthesis of results	21	Present results of each meta-analysis done, including confidence/credible intervals. *In larger networks, authors may focus on comparisons versus a particular comparator (e.g. placebo or standard care), with full findings presented in an appendix. League tables and forest plots may be considered to summarize pairwise comparisons.* If additional summary measures were explored (such as treatment rankings), these should also be presented.	**Figure S2, S3**
**Exploration for inconsistency**	**S5**	Describe results from investigations of inconsistency. This may include such information as measures of model fit to compare consistency and inconsistency models, *P* values from statistical tests, or summary of inconsistency estimates from different parts of the treatment network.	9
Risk of bias across studies	22	Present results of any assessment of risk of bias across studies for the evidence base being studied.	**9**
Results of additional analyses	23	Give results of additional analyses, if done (e.g., sensitivity or subgroup analyses, meta-regression analyses*, alternative network geometries studied, alternative choice of prior distributions for Bayesian analyses,* and so forth).	**10-14**
**DISCUSSION**			
Summary of evidence	24	Summarize the main findings, including the strength of evidence for each main outcome; consider their relevance to key groups (e.g., healthcare providers, users, and policy-makers).	**15-19**

Limitations	25	Discuss limitations at study and outcome level (e.g., risk of bias), and at review level (e.g., incomplete retrieval of identified research, reporting bias). *Comment on the validity of the assumptions, such as transitivity and consistency. Comment on any concerns regarding network geometry (e.g., avoidance of certain comparisons).*	**19-20**
Conclusions	26	Provide a general interpretation of the results in the context of other evidence, and implications for future research.	**20**
**FUNDING**			
Funding	27	Describe sources of funding for the systematic review and other support (e.g., supply of data); role of funders for the systematic review. This should also include information regarding whether funding has been received from manufacturers of treatments in the network and/or whether some of the authors are content experts with professional conflicts of interest that could affect use of treatments in the network.	**20**

PICOS = population, intervention, comparators, outcomes, study design. * Text in italics depicts words specific to network meta-analyses to guide the PRISMA statement. † Authors used appendices to present detailed relevant information for items in this section.

Table S2. Search strategy

Search strategy for PubMed, EMBASE, and Cochrane Library databases	
**PubMed**	((((((((((((non-small-cell lung cancer[Title/Abstract]) OR (non-small cell lung cancer[Title/Abstract])) OR (non small-cell lung cancer[Title/Abstract])) OR (non small cell lung cancer[Title/Abstract])) OR (non-small-cell lung carcinoma[Title/Abstract])) OR (non-small cell lung carcinoma[Title/Abstract])) OR (non small-cell lung carcinoma[Title/Abstract])) OR (non small cell lung carcinoma[Title/Abstract])) OR (nsclc[Title/Abstract])) AND ((epidermal growth factor receptor[Title/Abstract]) OR (EGFR[Title/Abstract]))) AND ((((((((((((((((((((treatment[Title/Abstract]) OR (therapy[Title/Abstract])) OR (tyrosine kinase inhibitor[Title/Abstract])) OR (TKI[Title/Abstract])) OR (osimertinib[Title/Abstract])) OR (dacomitinib[Title/Abstract])) OR (afatinib[Title/Abstract])) OR (erlotinib[Title/Abstract])) OR (gefitinib[Title/Abstract])) OR (icotinib[Title/Abstract])) OR (chemotherapy[Title/Abstract])) OR (furmonertinib[Title/Abstract])) OR (aumolertinib[Title/Abstract])) OR (amivantamab[Title/Abstract])) OR (lazertinib[Title/Abstract])) OR (zorifertinib[Title/Abstract])) OR (befotertinib[Title/Abstract])) OR (first-line[Title/Abstract])) OR (first line[Title/Abstract])) OR (treatment-naive[Title/Abstract])) OR (treatment-naïve[Title/Abstract])) OR (untreated[Title/Abstract]))) AND ((((((compare[Title/Abstract]) OR (comparison[Title/Abstract])) OR (comparative[Title/Abstract])) OR (comparing[Title/Abstract])) OR (versus[Title/Abstract])) OR (vs[Title/Abstract]))) AND (((((((Randomized Controlled Trial[Title/Abstract]) OR (controlled clinical trial[Title/Abstract])) OR (randomized[Title/Abstract])) OR (randomised[Title/Abstract])) OR (randomly[Title/Abstract])) OR (trial[Title/Abstract])) OR (phase[Title/Abstract]))) AND (English[Language])
**Web of science**	TS= (non-small-cell lung cancer OR non-small cell lung cancer OR non small-cell lung cancer OR non small cell lung cancer OR non-small-cell lung carcinoma OR non-small cell lung carcinoma OR non small-cell lung carcinoma OR non small cell lung carcinoma OR nsclc)<br>TS= (epidermal growth factor receptor OR EGFR)<br>TS= (treatment OR therapy OR tyrosine kinase inhibitor OR TKI OR osimertinib OR dacomitinib OR afatinib OR erlotinib OR gefitinib OR icotinib OR chemotherapy OR furmonertinib OR aumolertinib OR amivantamab OR lazertinib OR zorifertinib OR befotertinib OR first-line OR first line OR treatment-naive OR treatment-naïve OR untreated)<br>TS= (compare OR comparison OR comparative OR comparing OR versus OR vs)

	TS= (Randomized Controlled Trial OR controlled clinical trial OR randomized OR randomised OR randomly OR trial OR phase)<br>TS= (English)
**Cochrane**	#1(non-small-cell lung cancer OR non-small cell lung cancer OR non small-cell lung cancer OR non small cell lung cancer OR non-small-cell lung carcinoma OR non-small cell lung carcinoma OR non small-cell lung carcinoma OR non small cell lung carcinoma OR nsclc): ti,ab,kw<br>#2(epidermal growth factor receptor OR EGFR): ti,ab,kw<br>#3 (treatment OR therapy OR tyrosine kinase inhibitor OR TKI OR osimertinib OR dacomitinib OR afatinib OR erlotinib OR gefitinib OR icotinib OR chemotherapy OR furmonertinib OR aumolertinib OR amivantamab OR lazertinib OR zorifertinib OR befotertinib OR first-line OR first line OR treatment-naive OR treatment-naïve OR untreated): ti,ab,kw<br>#4(compare OR comparison OR comparative OR comparing OR versus OR vs): ti,ab,kw<br>#5(Randomized Controlled Trial OR controlled clinical trial OR randomized OR randomised OR randomly OR trial OR phase): ti,ab,kw<br>#6 (English): ti,ab,kw<br>#1and#2and#3and#4and#5and#6
**EMBASE**	#1 ‘non-small-cell lung cancer’:ab,ti OR ‘non-small cell lung cancer’:ab,ti OR ‘non small-cell lung cancer’:ab,ti OR ‘non small cell lung cancer’:ab,ti OR ‘non-small-cell lung carcinoma’:ab,ti OR ‘non-small cell lung carcinoma’:ab,ti OR ‘non small-cell lung carcinoma’:ab,ti OR ‘non small cell lung carcinoma’:ab,ti OR ‘nsclc’:ab,ti<br>#2 ‘epidermal growth factor receptor’:ab,ti OR ‘EGFR’:ab,ti<br>#3 ‘treatment’:ab,ti OR ‘therapy’:ab,ti OR ‘tyrosine kinase inhibitor’:ab,ti OR ‘TKI’:ab,ti OR ‘osimertinib’:ab,ti OR ‘dacomitinib’:ab,ti OR ‘afatinib’:ab,ti OR ‘erlotinib’:ab,ti OR ‘gefitinib’:ab,ti OR ‘icotinib’:ab,ti OR ‘chemotherapy’:ab,ti OR ‘furmonertinib’:ab,ti OR ‘aumolertinib’:ab,ti OR ‘amivantamab’:ab,ti OR ‘lazertinib’:ab,ti OR ‘zorifertinib’:ab,ti OR ‘befotertinib’:ab,ti OR ‘first-line’:ab,ti OR ‘first line’:ab,ti OR ‘treatment-naive’:ab,ti OR ‘treatment-naïve’:ab,ti OR ‘untreated’:ab,ti<br>#4 ‘compare’:ab,ti OR ‘comparison’:ab,ti OR ‘comparative’:ab,ti OR ‘comparing’:ab,ti OR ‘versus’:ab,ti OR ‘vs’:ab,ti<br>#5 ‘Randomized Controlled Trial’:ab,ti OR ‘controlled clinical trial’:ab,ti OR ‘randomized’:ab,ti OR ‘randomised’:ab,ti OR ‘randomly’:ab,ti OR ‘trial’:ab,ti OR ‘phase’:ab,ti<br>#6 ‘English’:ab,ti

Figure S1 Network map comparing different treatment outcomes in different groups

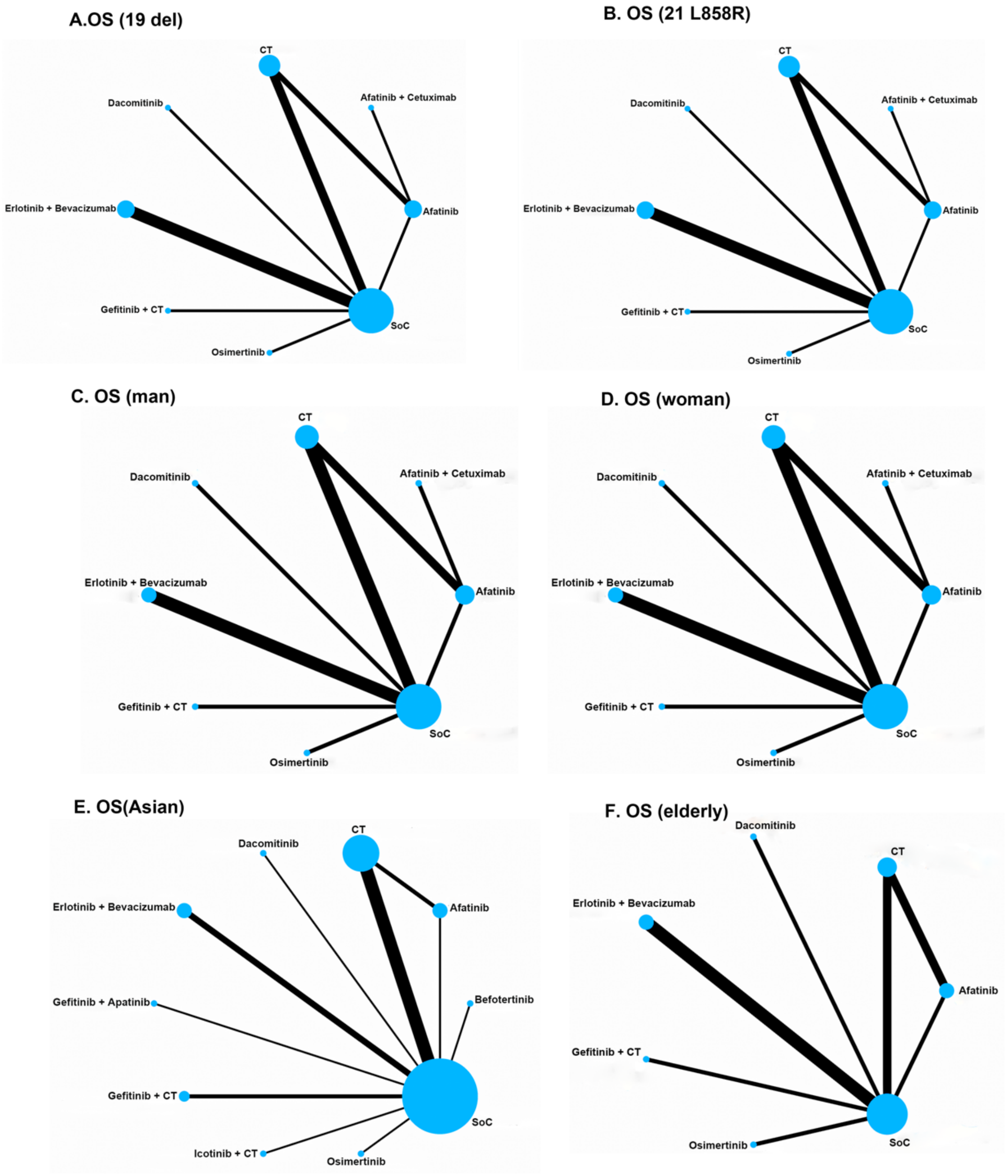

Each circular node represents a type of treatment. The node size is proportional to the total number of patients administering a treatment. Each line represents a type of head-to-head comparison. The width of lines is proportional to the total number of studies comparing the connected treatments. CT, Chemotherapy; SoC, standard of care.

Figure S2 Heterogeneity analysis of PFS (All patients)

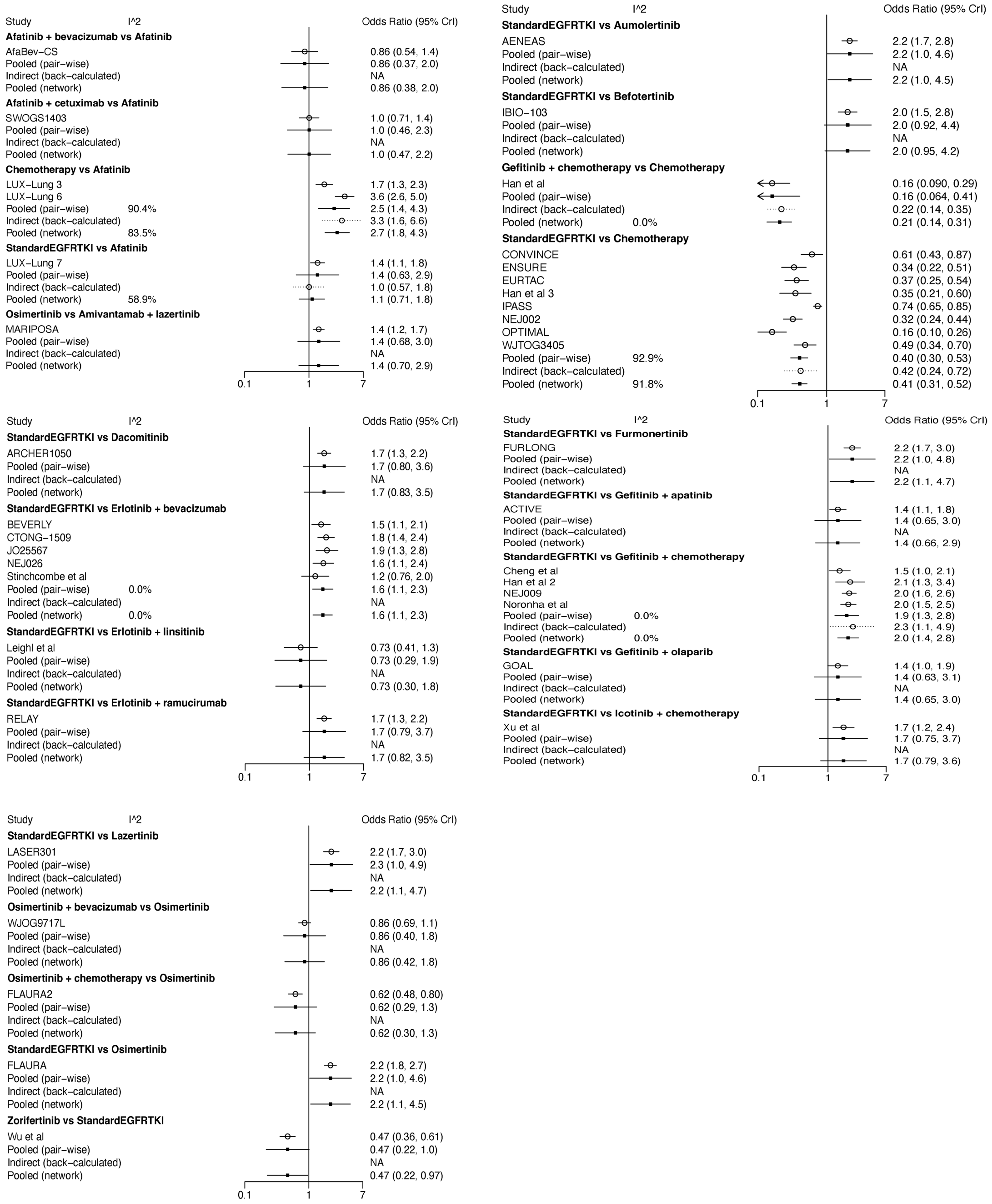

Values of I^2 below 25%, between 25% and 50% and above 50% represent low, medium and high heterogeneity, respectively.

Figure S3 Heterogeneity analysis of PFS based on different EGFR mutation types

EGFR 19 del

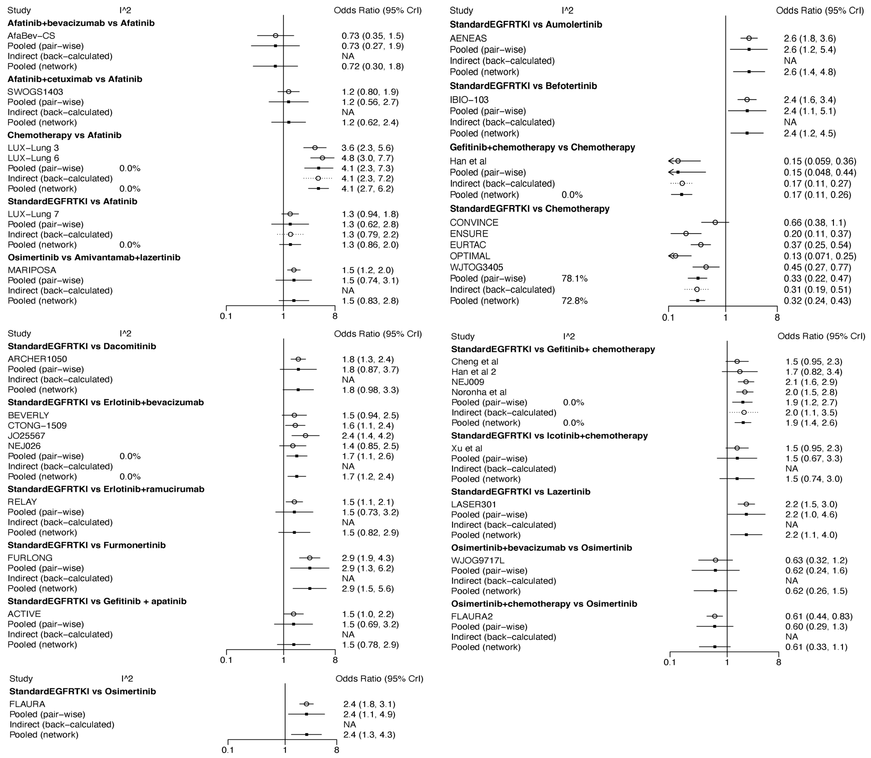

EGFR L858R

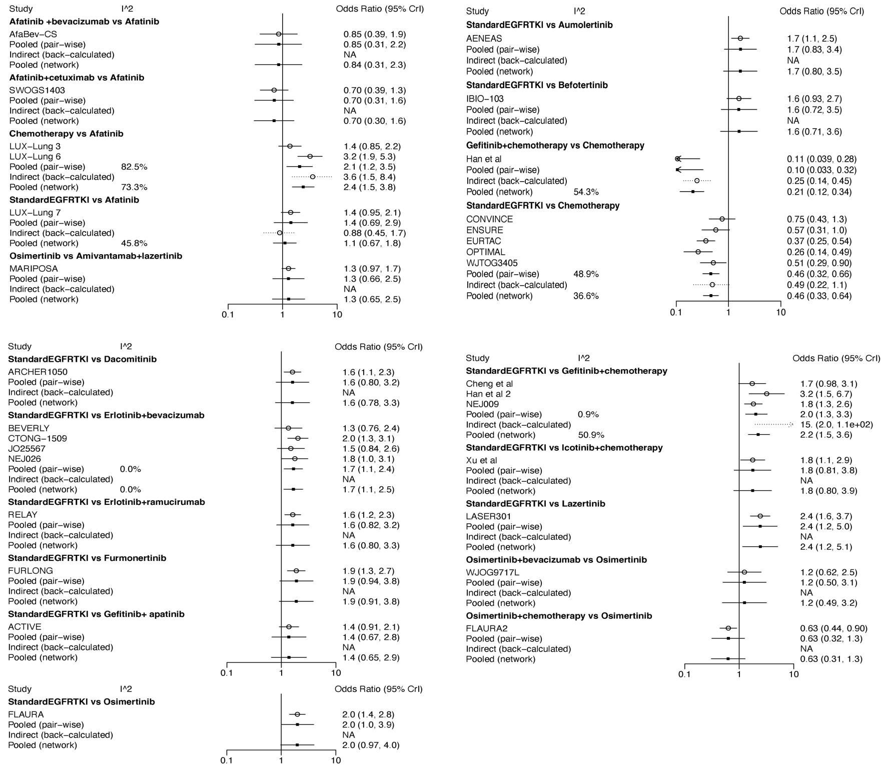

Figure S4 Heterogeneity analysis of PFS based on sex

Man

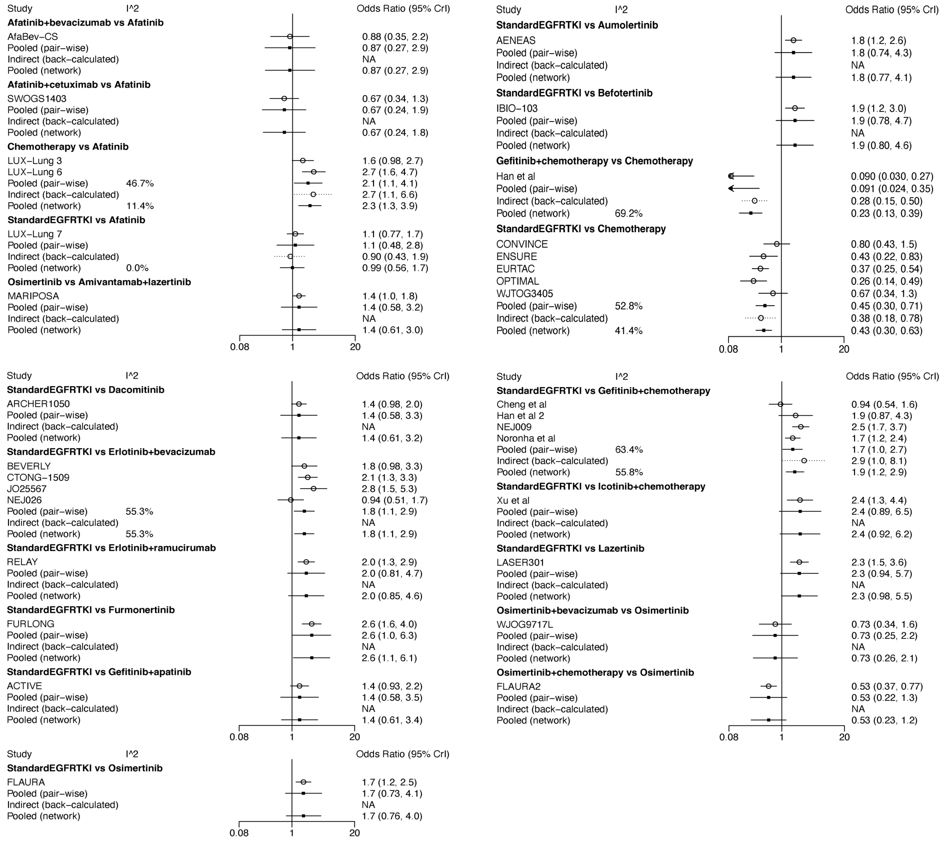

Woman

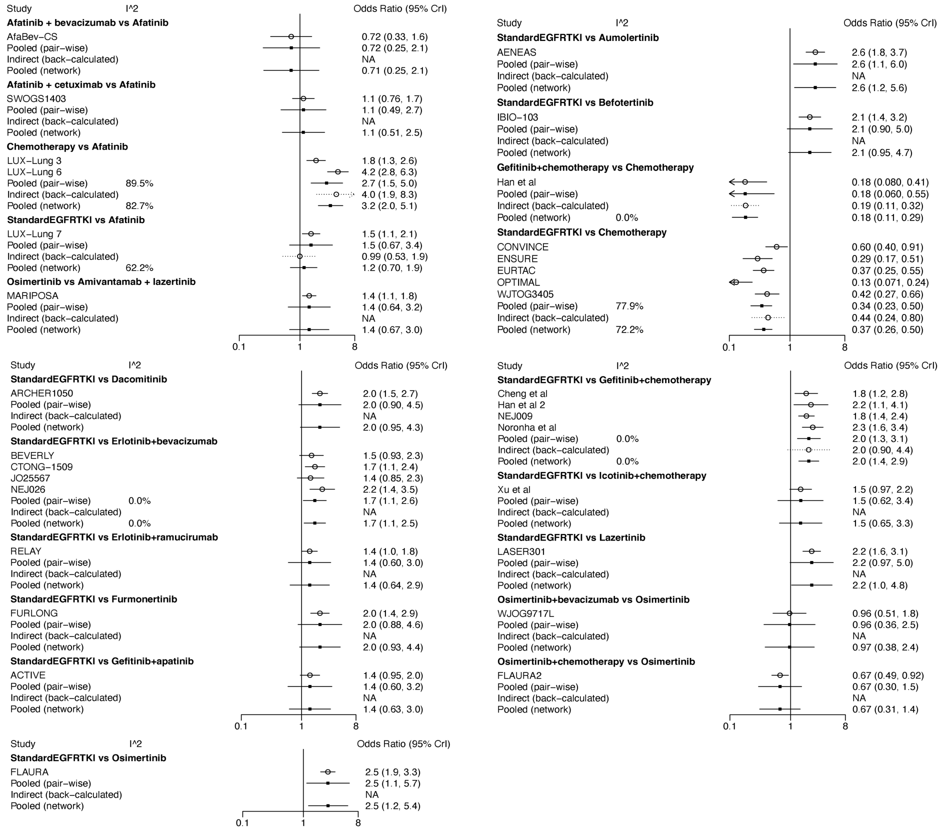

Figure S5 Heterogeneity analysis of PFS based on age and race

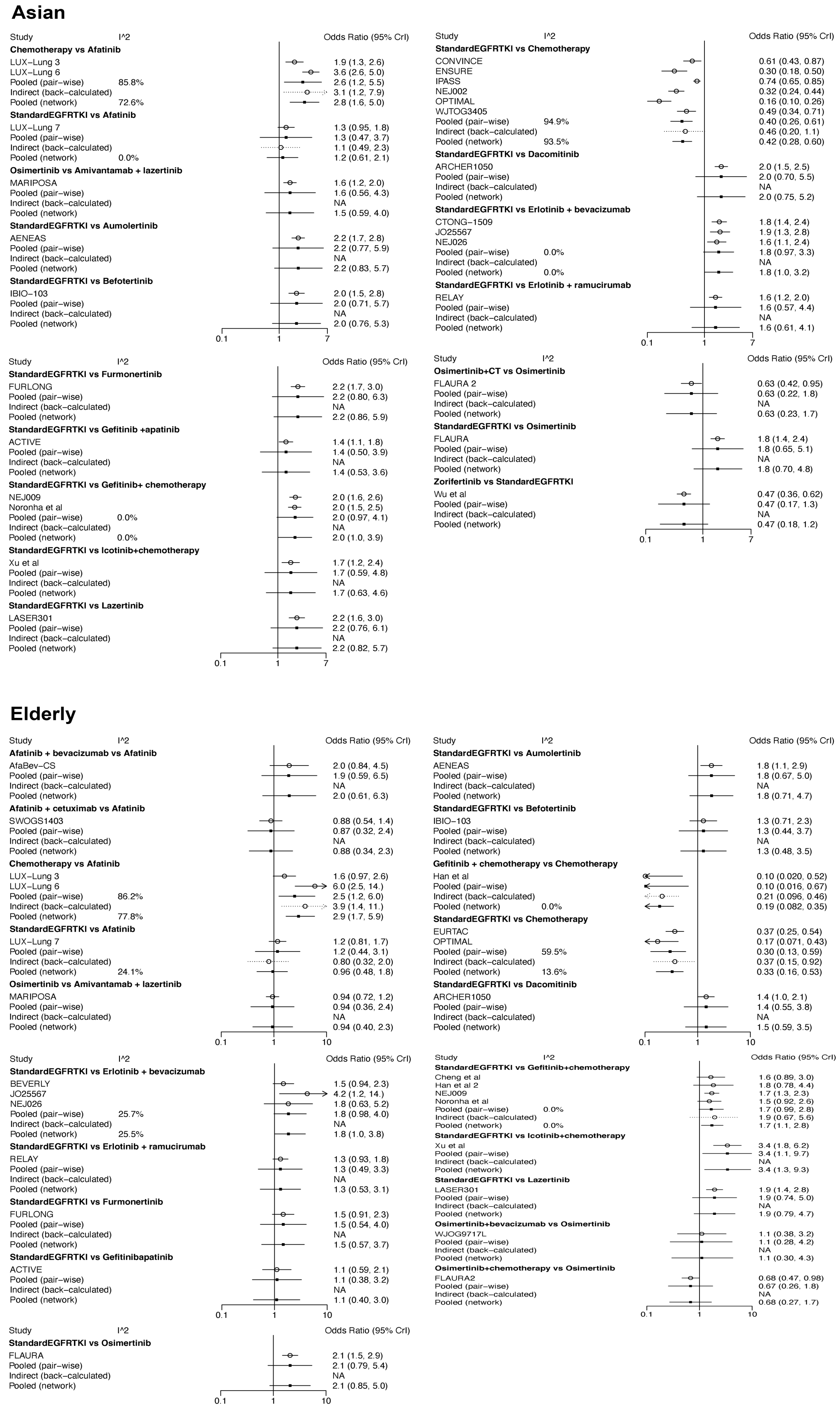

Figure S6 Heterogeneity analysis of OS based on all patients and different EGFR mutation types

OS (All patients)

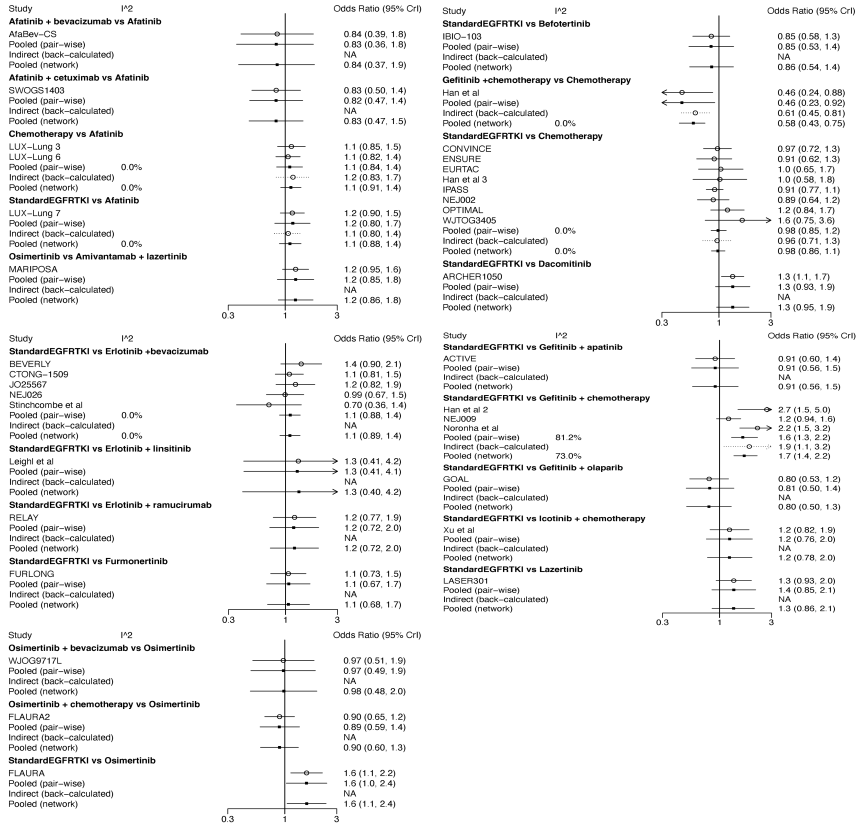

EGFR 19 del

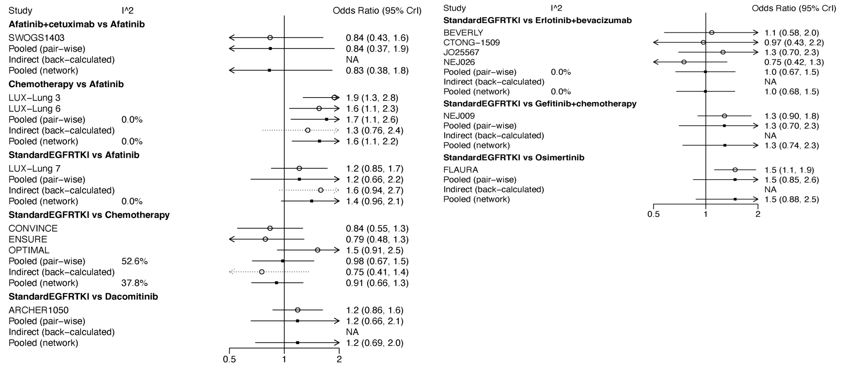

EGFR L858R

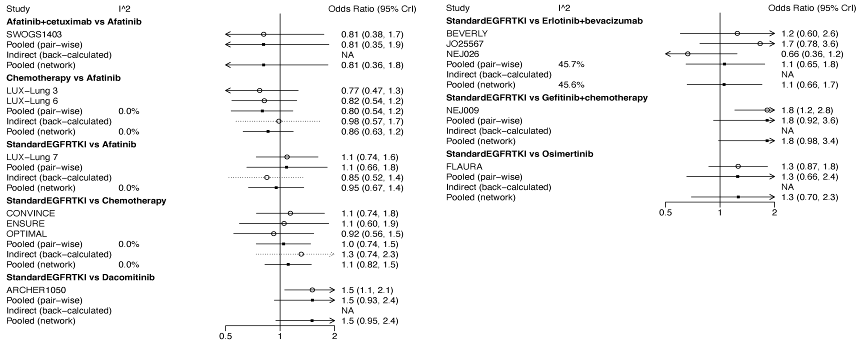

Figure S7 Heterogeneity analysis of OS based on sex, age and race

Man

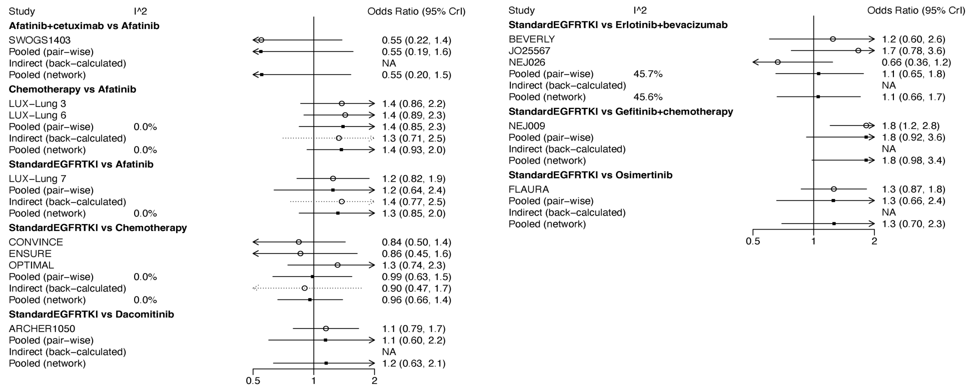

Woman

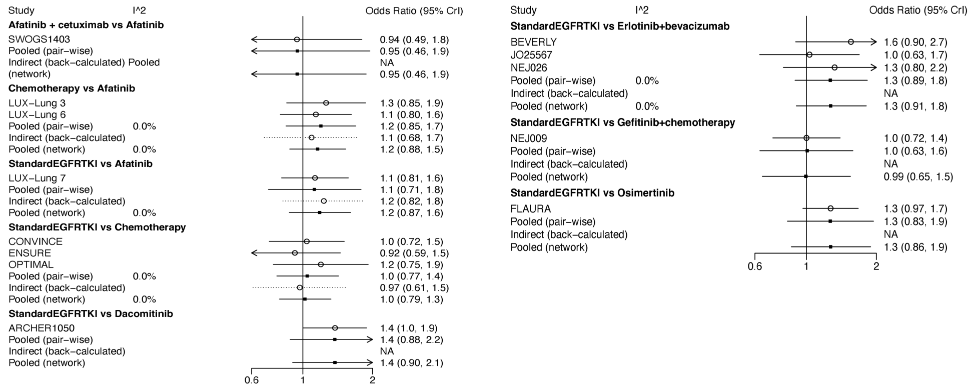

Asian

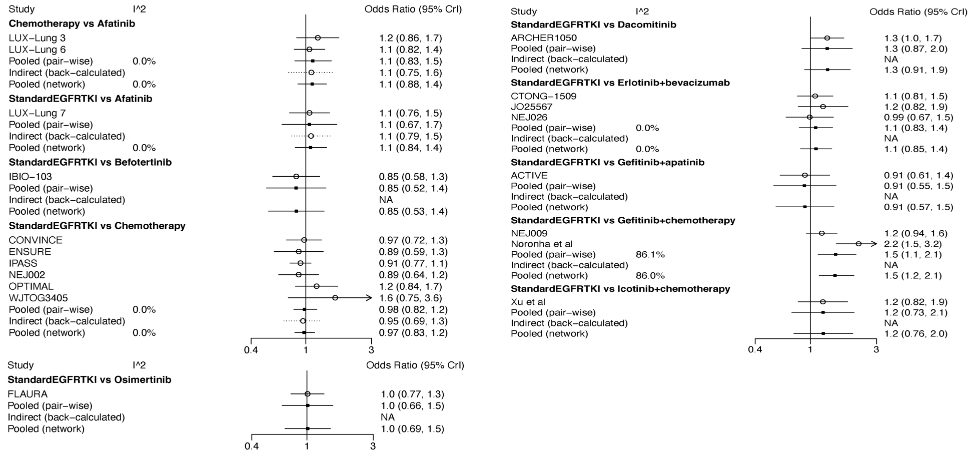

Elderly

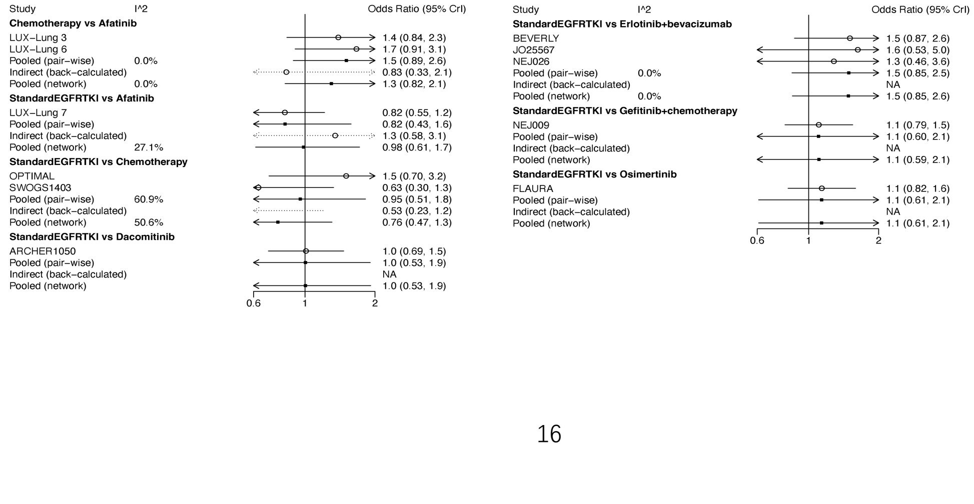

Figure S8 Inconsistency analysis of PFS

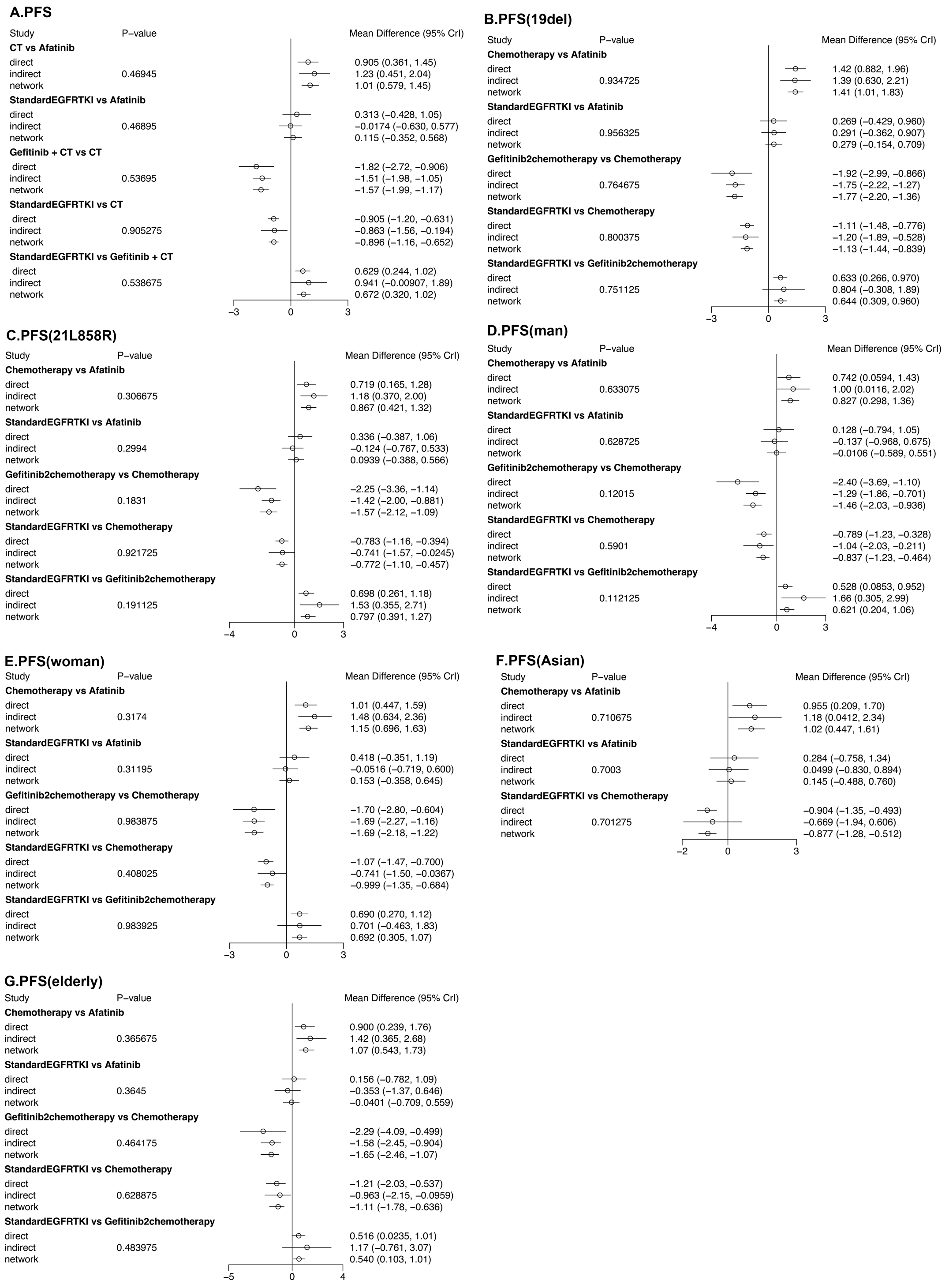

P value > 0.05 indicates that there is no inconsistency.

Figure S9 Inconsistency analysis of OS

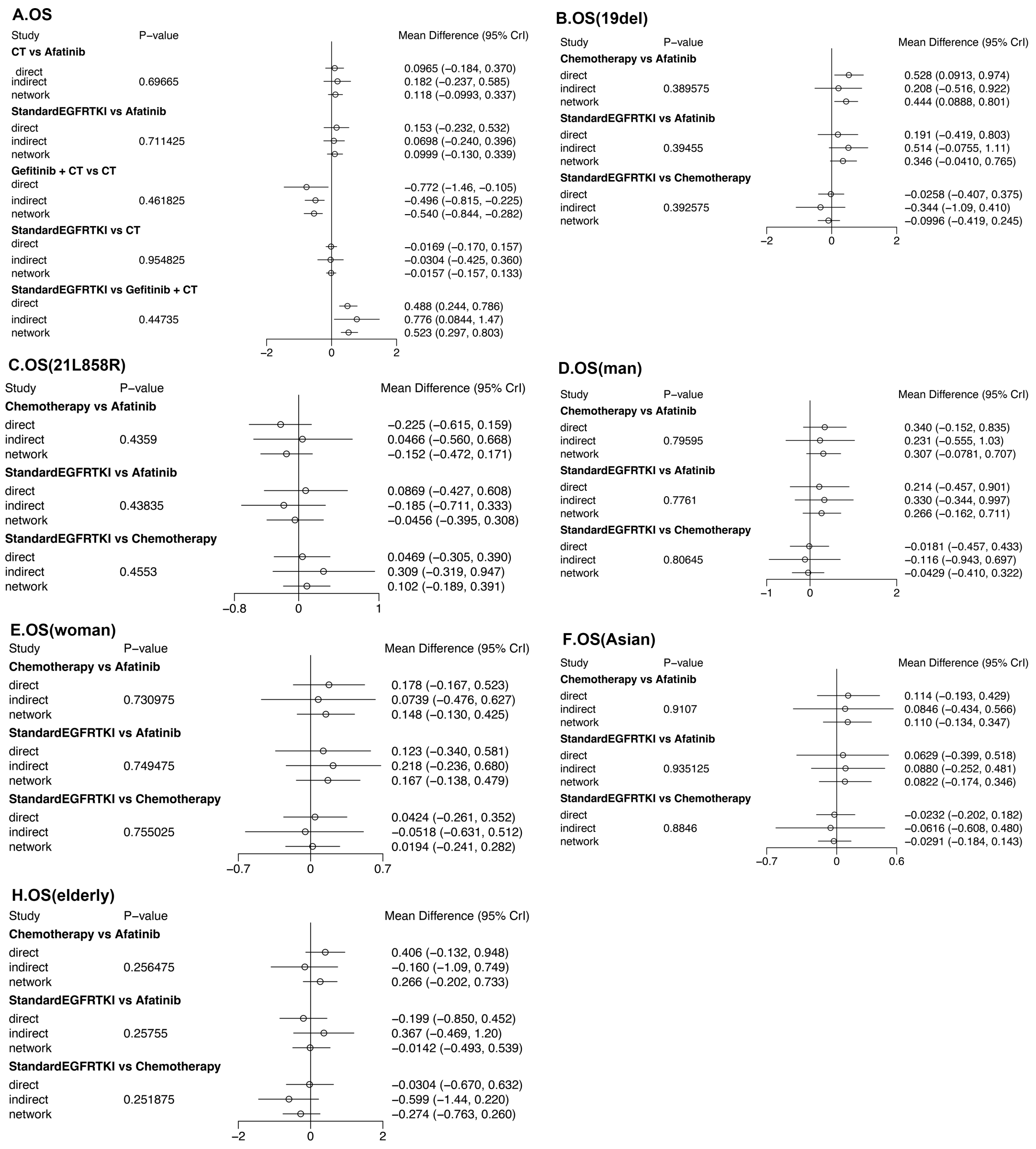

P value > 0.05 indicates that there is no inconsistency.

Figure S10 Pooled estimates of the network meta-analysis

A. OS(19del)

**Afa**	0.84 (0.38, 1.87)	**1.56 (1.09, 2.22)**	1.19 (0.61, 2.38)	1.42 (0.83, 2.5)	1.1 (0.56, 2.24)	0.96 (0.5, 1.87)	1.41 (0.96, 2.13)
1.19 (0.54, 2.61)	**Afa + Cet**	1.86 (0.77, 4.41)	1.42 (0.5, 4.04)	1.68 (0.65, 4.46)	1.31 (0.46, 3.77)	1.14 (0.41, 3.19)	1.68 (0.69, 4.1)
**0.64 (0.45, 0.91)**	0.54 (0.23, 1.3)	**CT**	0.76 (0.41, 1.47)	0.91 (0.55, 1.52)	0.7 (0.37, 1.38)	0.61 (0.33, 1.15)	0.9 (0.66, 1.27)
0.84 (0.42, 1.63)	0.71 (0.25, 1.99)	1.31 (0.68, 2.45)	**Dac**	1.19 (0.61, 2.32)	0.92 (0.42, 2.04)	0.81 (0.37, 1.72)	1.18 (0.68, 2.05)
0.7 (0.4, 1.21)	0.6 (0.22, 1.55)	1.1 (0.66, 1.81)	0.84 (0.43, 1.64)	**Erl + Bev**	0.78 (0.39, 1.54)	0.68 (0.35, 1.29)	1 (0.68, 1.46)
0.91 (0.45, 1.8)	0.77 (0.27, 2.18)	1.42 (0.72, 2.71)	1.08 (0.49, 2.39)	1.29 (0.65, 2.56)	**Gef + CT**	0.87 (0.4, 1.88)	1.28 (0.73, 2.27)
1.04 (0.54, 1.99)	0.88 (0.31, 2.44)	1.63 (0.87, 2.99)	1.24 (0.58, 2.67)	1.48 (0.77, 2.84)	1.14 (0.53, 2.5)	**Osi**	1.47 (0.87, 2.5)
0.71 (0.47, 1.04)	0.6 (0.24, 1.44)	1.11 (0.79, 1.52)	0.85 (0.49, 1.46)	1 (0.69, 1.47)	0.78 (0.44, 1.38)	0.68 (0.4, 1.14)	**SoC**

B. OS(21 L858R)

**Afa**	0.81 (0.36, 1.83)	0.86 (0.62, 1.19)	0.64 (0.35, 1.14)	0.71 (0.42, 1.17)	0.86 (0.47, 1.55)	0.96 (0.54, 1.71)	0.96 (0.67, 1.36)
1.23 (0.55, 2.77)	**Afa + Cet**	1.06 (0.44, 2.55)	0.78 (0.29, 2.13)	0.88 (0.33, 2.26)	1.06 (0.39, 2.88)	1.18 (0.44, 3.16)	1.18 (0.49, 2.87)
1.16 (0.84, 1.61)	0.94 (0.39, 2.26)	**CT**	0.74 (0.42, 1.27)	0.82 (0.51, 1.31)	1 (0.56, 1.74)	1.11 (0.65, 1.91)	1.11 (0.82, 1.49)
1.57 (0.88, 2.86)	1.28 (0.47, 3.44)	1.36 (0.78, 2.36)	**Dac**	1.12 (0.61, 2.01)	1.35 (0.69, 2.69)	1.51 (0.79, 2.89)	1.51 (0.94, 2.41)
1.41 (0.86, 2.35)	1.14 (0.44, 3.01)	1.22 (0.76, 1.94)	0.89 (0.5, 1.63)	**Erl + Bev**	1.21 (0.66, 2.19)	1.35 (0.76, 2.42)	1.35 (0.94, 1.94)
1.16 (0.64, 2.14)	0.95 (0.35, 2.59)	1 (0.57, 1.79)	0.74 (0.37, 1.45)	0.82 (0.46, 1.51)	**Gef + CT**	1.12 (0.58, 2.18)	1.11 (0.69, 1.81)
1.04 (0.59, 1.86)	0.85 (0.32, 2.3)	0.9 (0.52, 1.55)	0.66 (0.35, 1.27)	0.74 (0.41, 1.31)	0.9 (0.46, 1.73)	**Osi**	1 (0.63, 1.57)
1.04 (0.73, 1.5)	0.85 (0.35, 2.05)	0.9 (0.67, 1.21)	0.66 (0.42, 1.06)	0.74 (0.52, 1.07)	0.9 (0.55, 1.45)	1 (0.64, 1.58)	**SoC**

C. OS(man)

**Afa**	0.55 (0.22, 1.39)	**1.36 (1.03, 1.81)**	1.13 (0.7, 1.84)	1.25 (0.75, 2.08)	0.72 (0.43, 1.2)	1.04 (0.64, 1.68)	1.3 (0.96, 1.78)
1.82 (0.72, 4.56)	**Afa + Cet**	2.48 (0.94, 6.52)	2.07 (0.72, 5.85)	2.28 (0.79, 6.54)	1.3 (0.45, 3.73)	1.88 (0.66, 5.32)	2.38 (0.89, 6.28)
**0.73 (0.55, 0.97)**	0.4 (0.15, 1.06)	**CT**	0.83 (0.52, 1.33)	0.92 (0.56, 1.5)	**0.53 (0.32, 0.87)**	0.76 (0.48, 1.21)	0.96 (0.72, 1.27)
0.88 (0.54, 1.43)	0.48 (0.17, 1.38)	1.2 (0.75, 1.92)	**Dac**	1.1 (0.64, 1.92)	0.63 (0.36, 1.1)	0.91 (0.54, 1.55)	1.15 (0.79, 1.67)
0.8 (0.48, 1.33)	0.44 (0.15, 1.26)	1.09 (0.67, 1.78)	0.91 (0.52, 1.57)	**Erl + Bev**	0.57 (0.32, 1.02)	0.83 (0.48, 1.43)	1.04 (0.69, 1.56)
1.4 (0.83, 2.34)	0.77 (0.27, 2.21)	**1.9 (1.15, 3.14)**	1.58 (0.91, 2.76)	1.75 (0.98, 3.11)	**Gef + CT**	1.45 (0.83, 2.52)	**1.82 (1.21, 2.76)**
0.97 (0.59, 1.56)	0.53 (0.19, 1.51)	1.32 (0.83, 2.09)	1.1 (0.65, 1.86)	1.21 (0.7, 2.1)	0.69 (0.4, 1.2)	**Osi**	1.26 (0.87, 1.83)
0.77 (0.56, 1.04)	0.42 (0.16, 1.12)	1.05 (0.79, 1.39)	0.87 (0.6, 1.26)	0.96 (0.64, 1.44)	**0.55 (0.36, 0.83)**	0.79 (0.55, 1.15)	**SoC**

D. OS(woman)

**Afa**	1.25 (0.81, 1.94)	1.11 (0.93, 1.33)	0.81 (0.58, 1.13)	0.98 (0.74, 1.3)	1.17 (0.74, 1.84)	**0.73 (0.55, 0.97)**	0.86 (0.55, 1.36)	1.06 (0.75, 1.48)	1.07 (0.88, 1.3)
0.8 (0.52, 1.24)	**Bef**	0.89 (0.59, 1.33)	0.64 (0.4, 1.04)	0.78 (0.5, 1.21)	0.93 (0.53, 1.65)	**0.58 (0.37, 0.9)**	0.69 (0.39, 1.22)	0.84 (0.52, 1.36)	0.85 (0.58, 1.26)
0.9 (0.75, 1.08)	1.13 (0.75, 1.69)	**CT**	**0.73 (0.54, 0.98)**	0.88 (0.7, 1.11)	1.05 (0.68, 1.61)	**0.65 (0.51, 0.83)**	0.78 (0.51, 1.19)	0.95 (0.71, 1.28)	0.96 (0.86, 1.08)
1.24 (0.89, 1.74)	1.55 (0.96, 2.51)	**1.38 (1.02, 1.85)**	**Dac**	1.21 (0.86, 1.71)	1.45 (0.88, 2.38)	0.9 (0.64, 1.27)	1.07 (0.66, 1.76)	1.31 (0.89, 1.93)	**1.32 (1.01, 1.74)**
1.02 (0.77, 1.35)	1.28 (0.83, 1.99)	1.14 (0.9, 1.44)	0.83 (0.59, 1.16)	**Erl + Bev**	1.2 (0.75, 1.89)	**0.74 (0.55, 0.99)**	0.89 (0.56, 1.4)	1.08 (0.77, 1.52)	1.09 (0.89, 1.34)
0.86 (0.54, 1.35)	1.07 (0.61, 1.89)	0.95 (0.62, 1.46)	0.69 (0.42, 1.13)	0.84 (0.53, 1.33)	**Gef + Apa**	**0.62 (0.39, 0.99)**	0.74 (0.41, 1.33)	0.9 (0.55, 1.48)	0.91 (0.6, 1.38)
**1.38 (1.03, 1.83)**	**1.72 (1.11, 2.69)**	**1.53 (1.2, 1.95)**	1.11 (0.79, 1.57)	**1.35 (1.01, 1.81)**	**1.61 (1.01, 2.56)**	**Gef + CT**	1.19 (0.75, 1.89)	**1.45 (1.03, 2.05)**	**1.47 (1.19, 1.81)**
1.16 (0.73, 1.82)	1.45 (0.82, 2.55)	1.28 (0.84, 1.97)	0.93 (0.57, 1.53)	1.13 (0.71, 1.79)	1.35 (0.75, 2.42)	0.84 (0.53, 1.33)	**Ico + CT**	1.22 (0.75, 2)	1.23 (0.82, 1.86)
0.95 (0.68, 1.33)	1.19 (0.74, 1.91)	1.05 (0.78, 1.42)	0.76 (0.52, 1.12)	0.93 (0.66, 1.3)	1.11 (0.67, 1.81)	**0.69 (0.49, 0.97)**	0.82 (0.5, 1.34)	**Osi**	1.01 (0.77, 1.33)
0.94 (0.77, 1.14)	1.17 (0.79, 1.74)	1.04 (0.93, 1.17)	**0.76 (0.58, 0.99)**	0.92 (0.75, 1.12)	1.09 (0.72, 1.66)	**0.68 (0.55, 0.84)**	0.81 (0.54, 1.22)	0.99 (0.75, 1.3)	**SoC**

E.OS(Asian)

**Afa**	1.25 (0.81, 1.94)	1.11 (0.93, 1.33)	0.81 (0.58, 1.13)	0.98 (0.74, 1.3)	1.17 (0.74, 1.84)	**0.73 (0.55, 0.97)**	0.86 (0.55, 1.36)	1.06 (0.75, 1.48)	1.07 (0.88, 1.3)
0.8 (0.52, 1.24)	**Bef**	0.89 (0.59, 1.33)	0.64 (0.4, 1.04)	0.78 (0.5, 1.21)	0.93 (0.53, 1.65)	**0.58 (0.37, 0.9)**	0.69 (0.39, 1.22)	0.84 (0.52, 1.36)	0.85 (0.58, 1.26)
0.9 (0.75, 1.08)	1.13 (0.75, 1.69)	**CT**	**0.73 (0.54, 0.98)**	0.88 (0.7, 1.11)	1.05 (0.68, 1.61)	**0.65 (0.51, 0.83)**	0.78 (0.51, 1.19)	0.95 (0.71, 1.28)	0.96 (0.86, 1.08)
1.24 (0.89, 1.74)	1.55 (0.96, 2.51)	**1.38 (1.02, 1.85)**	**Dac**	1.21 (0.86, 1.71)	1.45 (0.88, 2.38)	0.9 (0.64, 1.27)	1.07 (0.66, 1.76)	1.31 (0.89, 1.93)	**1.32 (1.01, 1.74)**
1.02 (0.77, 1.35)	1.28 (0.83, 1.99)	1.14 (0.9, 1.44)	0.83 (0.59, 1.16)	**Erl + Bev**	1.2 (0.75, 1.89)	**0.74 (0.55, 0.99)**	0.89 (0.56, 1.4)	1.08 (0.77, 1.52)	1.09 (0.89, 1.34)
0.86 (0.54, 1.35)	1.07 (0.61, 1.89)	0.95 (0.62, 1.46)	0.69 (0.42, 1.13)	0.84 (0.53, 1.33)	**Gef + Apa**	**0.62 (0.39, 0.99)**	0.74 (0.41, 1.33)	0.9 (0.55, 1.48)	0.91 (0.6, 1.38)
**1.38 (1.03, 1.83)**	**1.72 (1.11, 2.69)**	**1.53 (1.2, 1.95)**	1.11 (0.79, 1.57)	**1.35 (1.01, 1.81)**	**1.61 (1.01, 2.56)**	**Gef + CT**	1.19 (0.75, 1.89)	**1.45 (1.03, 2.05)**	**1.47 (1.19, 1.81)**
1.16 (0.73, 1.82)	1.45 (0.82, 2.55)	1.28 (0.84, 1.97)	0.93 (0.57, 1.53)	1.13 (0.71, 1.79)	1.35 (0.75, 2.42)	0.84 (0.53, 1.33)	**Ico + CT**	1.22 (0.75, 2)	1.23 (0.82, 1.86)
0.95 (0.68, 1.33)	1.19 (0.74, 1.91)	1.05 (0.78, 1.42)	0.76 (0.52, 1.12)	0.93 (0.66, 1.3)	1.11 (0.67, 1.81)	**0.69 (0.49, 0.97)**	0.82 (0.5, 1.34)	**Osi**	1.01 (0.77, 1.33)
0.94 (0.77, 1.14)	1.17 (0.79, 1.74)	1.04 (0.93, 1.17)	**0.76 (0.58, 0.99)**	0.92 (0.75, 1.12)	1.09 (0.72, 1.66)	**0.68 (0.55, 0.84)**	0.81 (0.54, 1.22)	0.99 (0.75, 1.3)	**SoC**

F. OS(elderly)

**Afa**	1.29 (0.92, 1.81)	0.94 (0.57, 1.55)	0.64 (0.37, 1.12)	0.86 (0.53, 1.38)	0.83 (0.52, 1.34)	0.95 (0.68, 1.33)
0.77 (0.55, 1.08)	**CT**	0.73 (0.43, 1.24)	**0.5 (0.28, 0.89)**	0.66 (0.4, 1.1)	0.64 (0.39, 1.07)	0.73 (0.5, 1.08)
1.06 (0.64, 1.76)	1.38 (0.8, 2.35)	**Dac**	0.68 (0.38, 1.22)	0.91 (0.55, 1.5)	0.89 (0.54, 1.46)	1.01 (0.7, 1.47)
1.56 (0.89, 2.73)	**2.02 (1.12, 3.63)**	1.47 (0.82, 2.62)	**Erl + Bev**	1.34 (0.77, 2.34)	1.3 (0.75, 2.27)	1.48 (0.95, 2.31)
1.16 (0.73, 1.87)	1.51 (0.91, 2.51)	1.09 (0.66, 1.8)	0.75 (0.43, 1.3)	**Gef + CT**	0.97 (0.61, 1.55)	1.11 (0.79, 1.54)
1.2 (0.75, 1.93)	1.55 (0.93, 2.58)	1.13 (0.69, 1.86)	0.77 (0.44, 1.34)	1.03 (0.64, 1.65)	**Osi**	1.14 (0.82, 1.59)
1.05 (0.75, 1.48)	1.36 (0.93, 2)	0.99 (0.68, 1.44)	0.67 (0.43, 1.05)	0.9 (0.65, 1.26)	0.88 (0.63, 1.22)	**SoC**

Figure S11 Ranking diagram for network meta-analysis of PFS

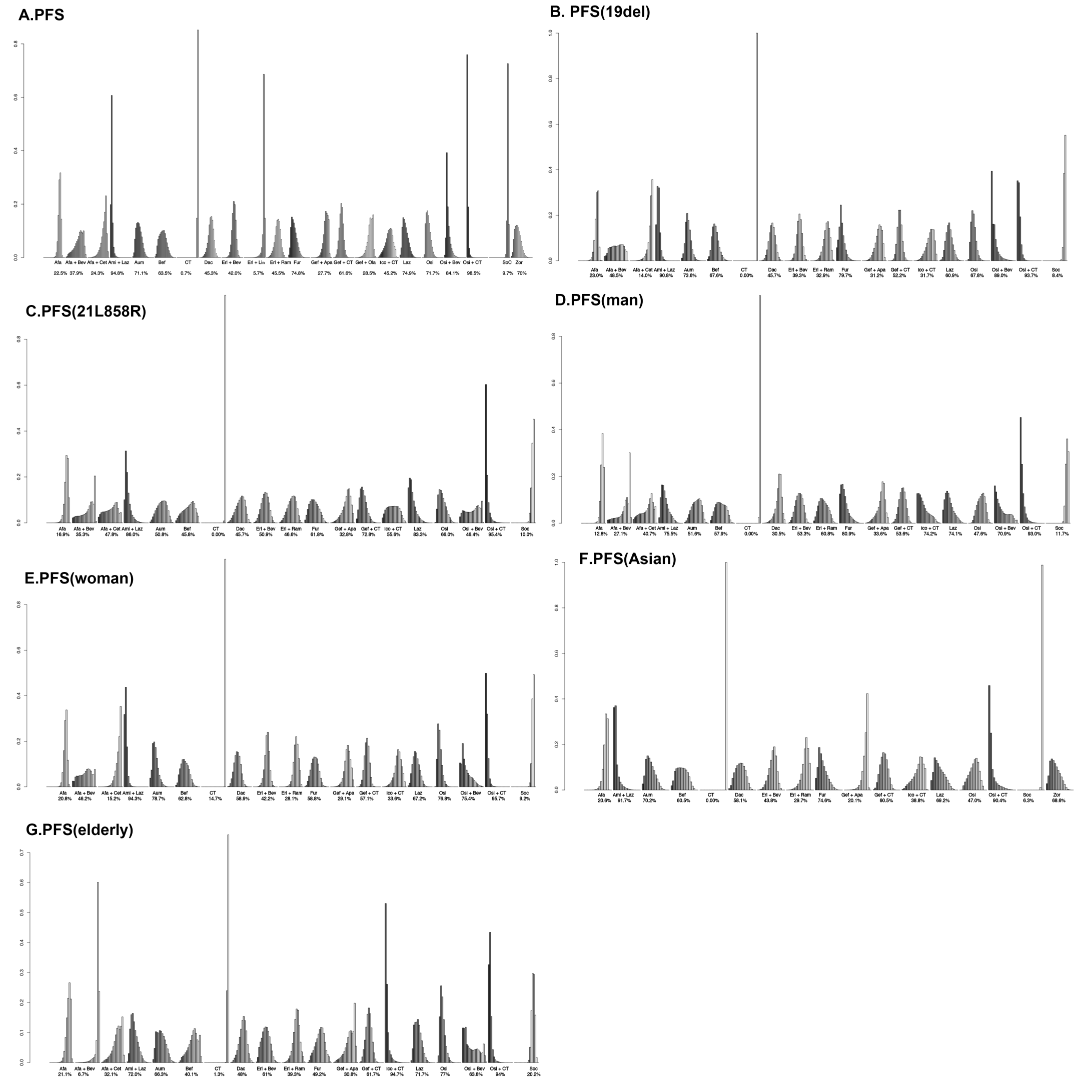

Figure S12 Ranking diagram for network meta-analysis of OS

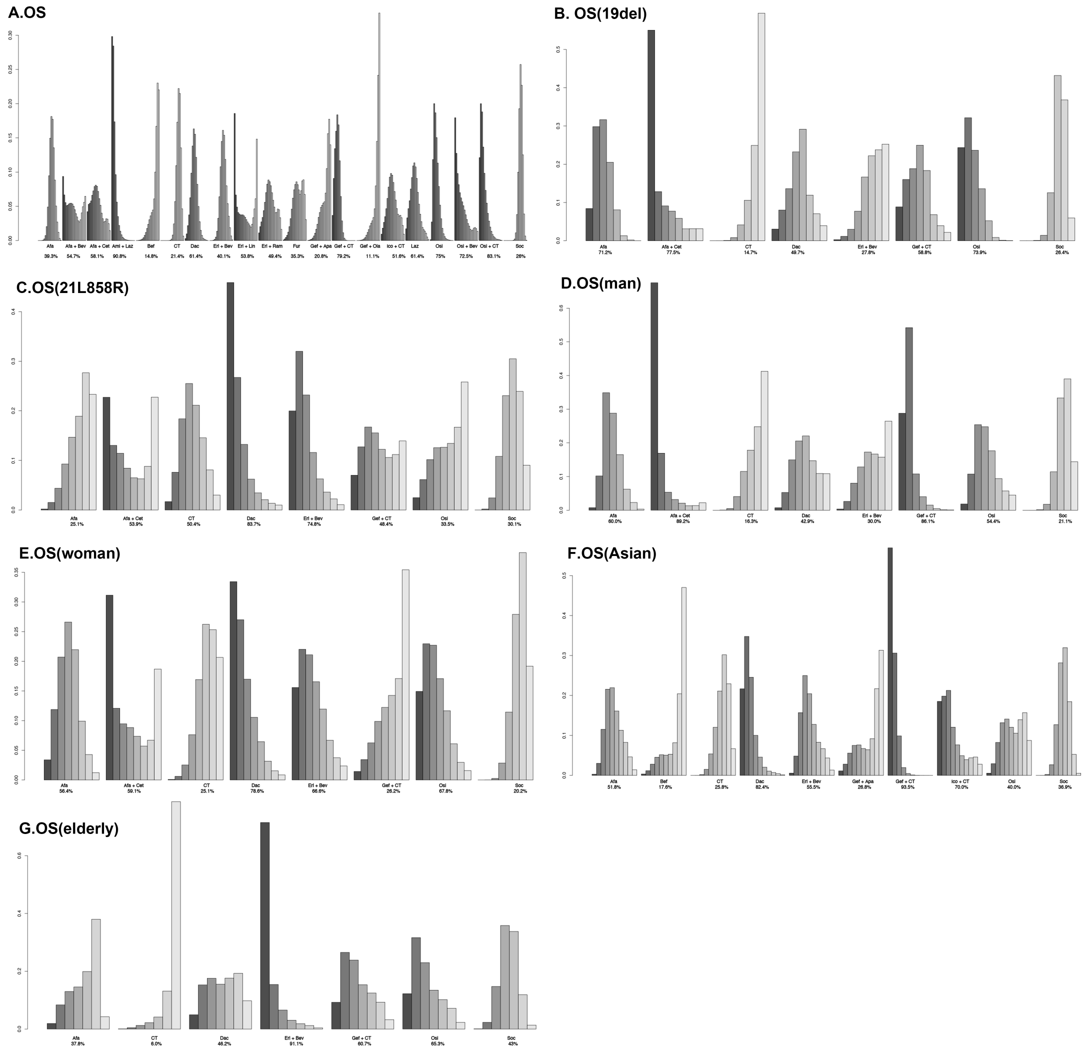

Figure S13 Brooks-Gelman-Rubin diagnostic plots for evaluating model convergence of PFS

A. PFS

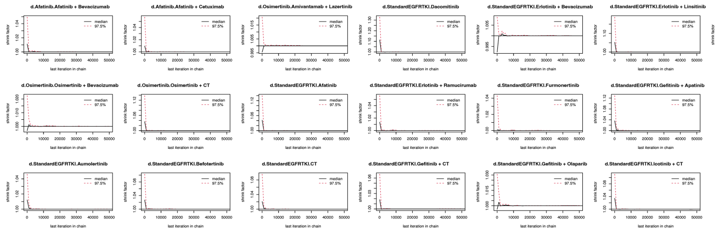

B. PFS(19del)

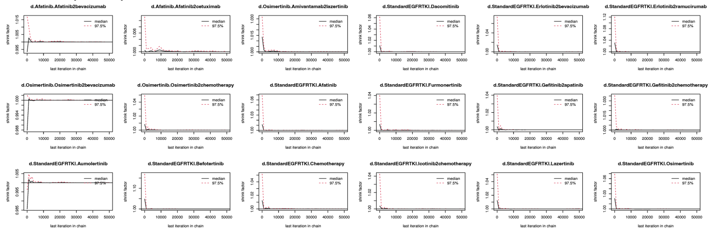

C.PFS(21L858R)

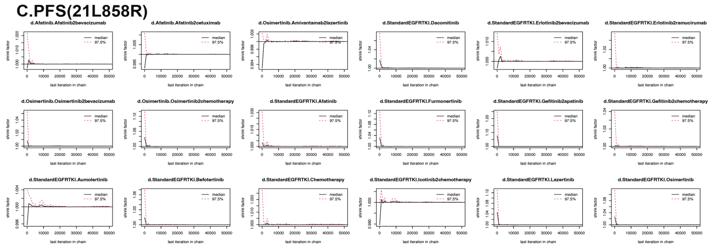

D.PFS(man)

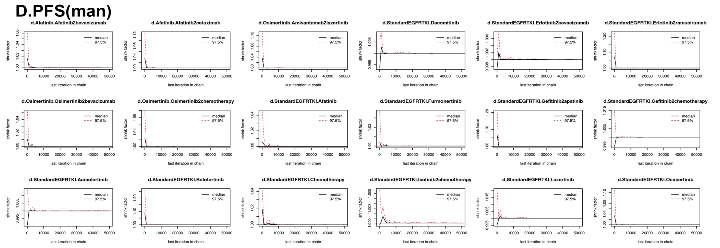

E.PFS(woman)

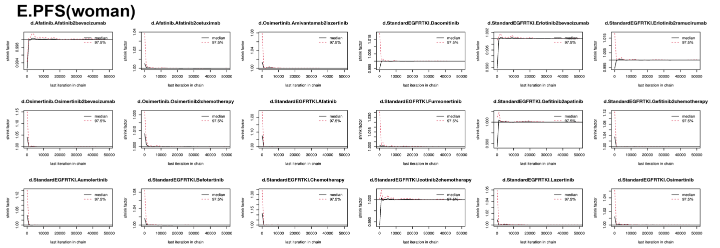

G.PFS(Asian)

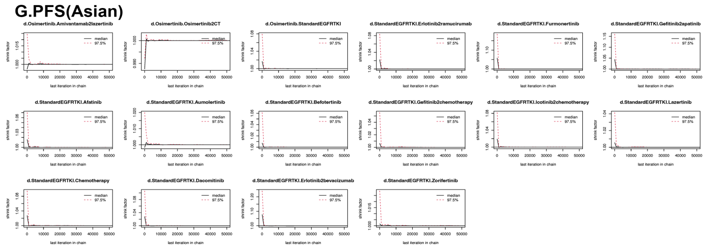

H.PFS(elderly)

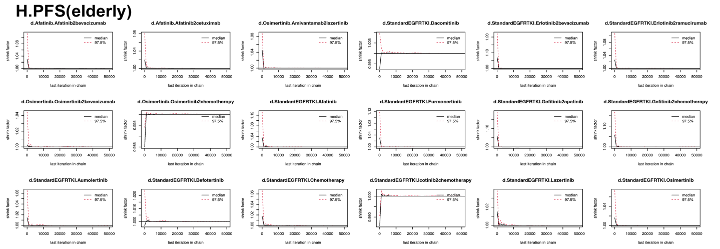

Figure S14 Brooks-Gelman-Rubin diagnostic plots for evaluating model convergence of OS

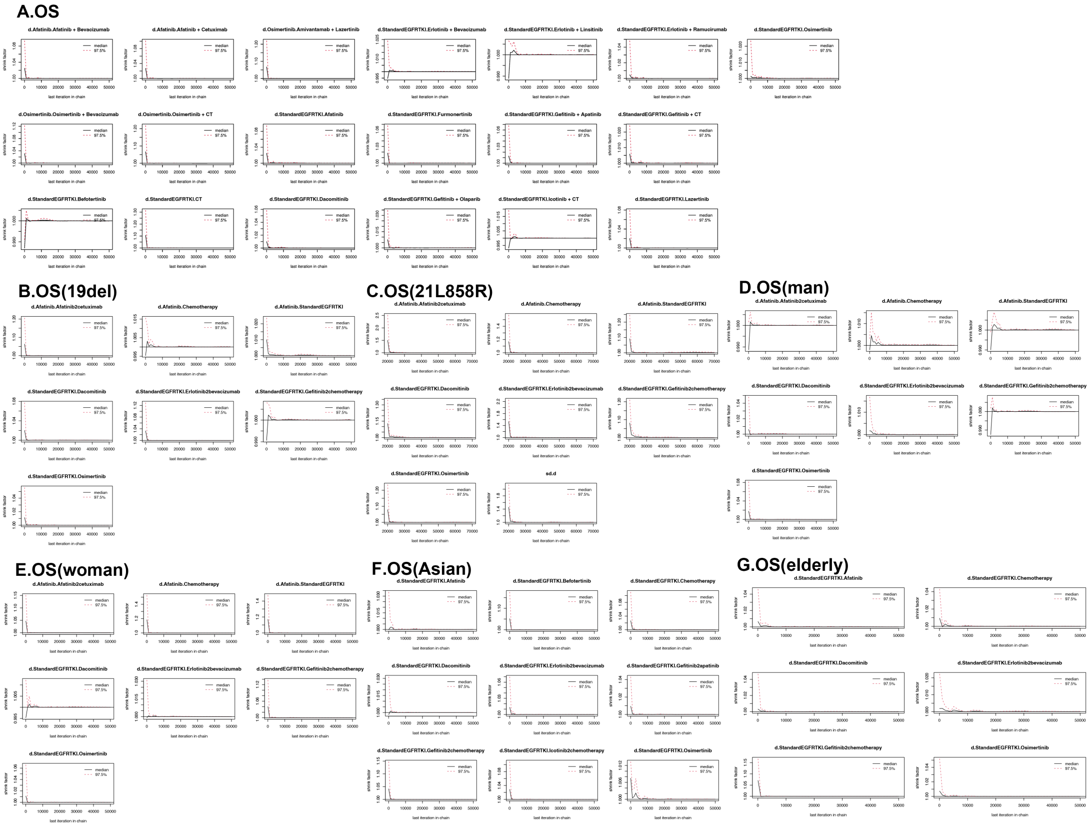

Figure S15 Trace plot for evaluating model convergence of PFS (all patients and EGFR mutations)

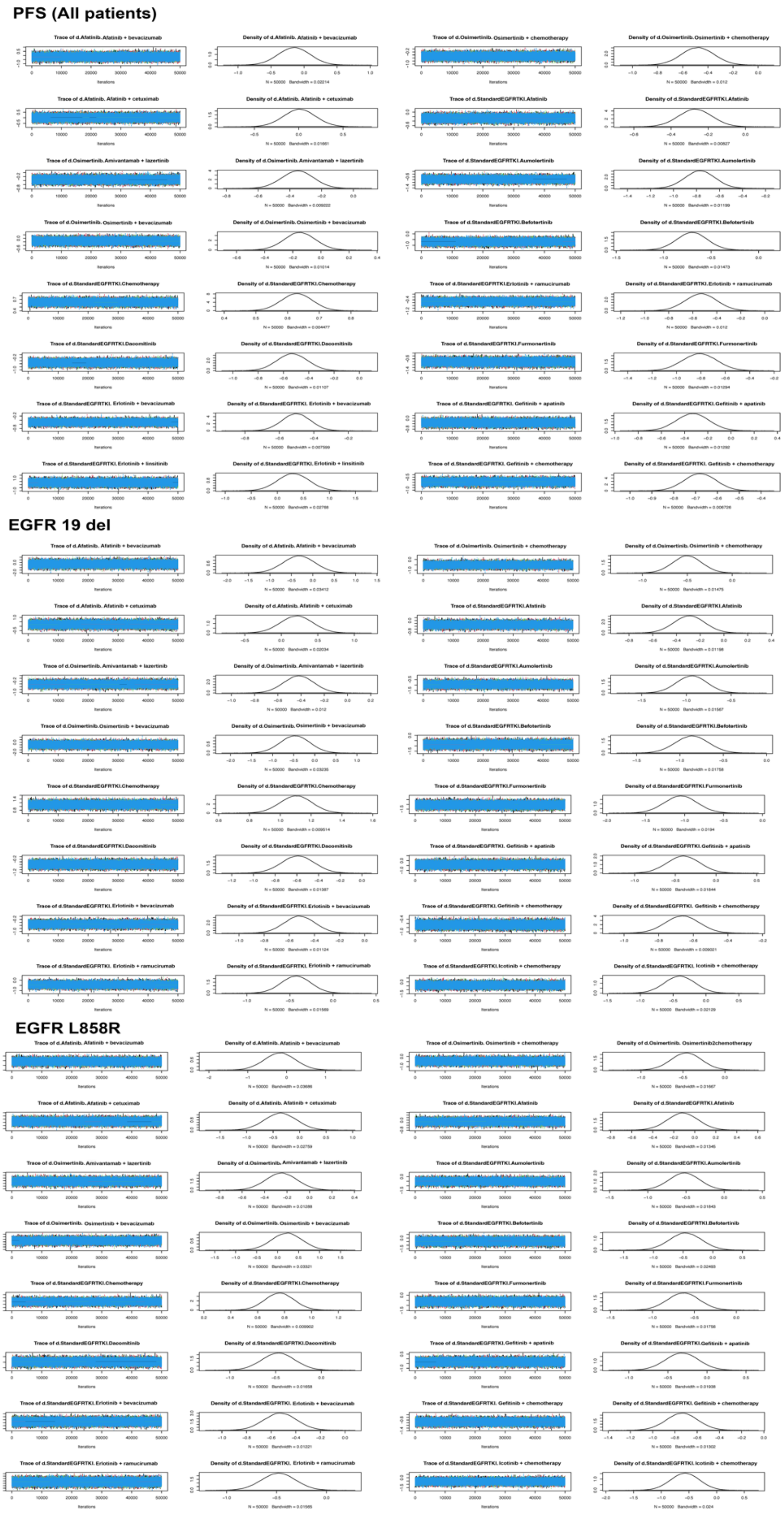

The overlapping area of MCMC chains accounts for most of the fluctuation range of the chains, and the fluctuation of a single chain cannot be identified and the distribution pattern of the density map is normal, which indicate that the convergence of the model is satisfactory

Figure S16 Trace plot for evaluating model convergence of PFS (Sex)

Man

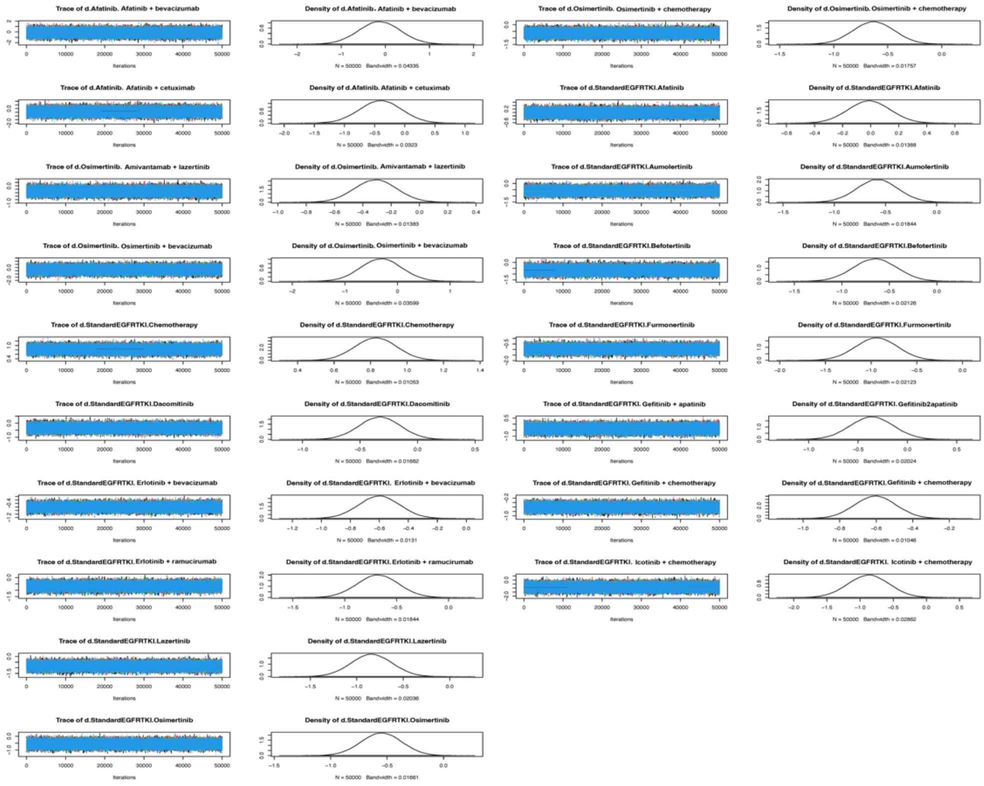

Woman

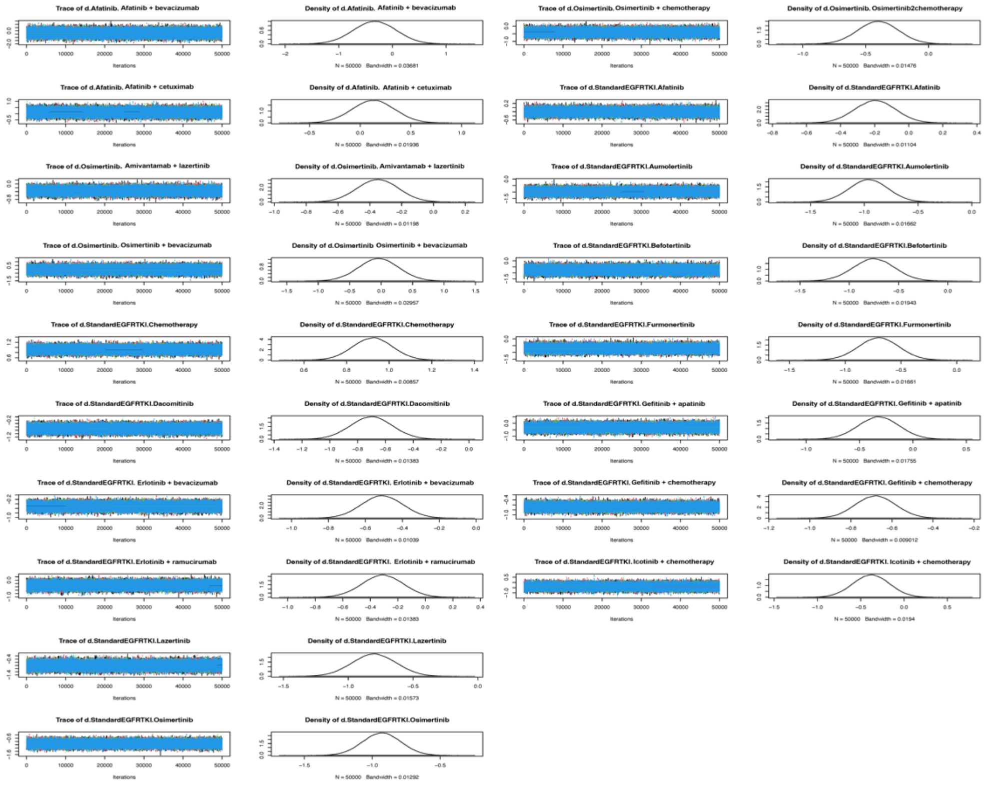

Figure S17 Trace plot for evaluating model convergence of PFS (age and race)

Asian

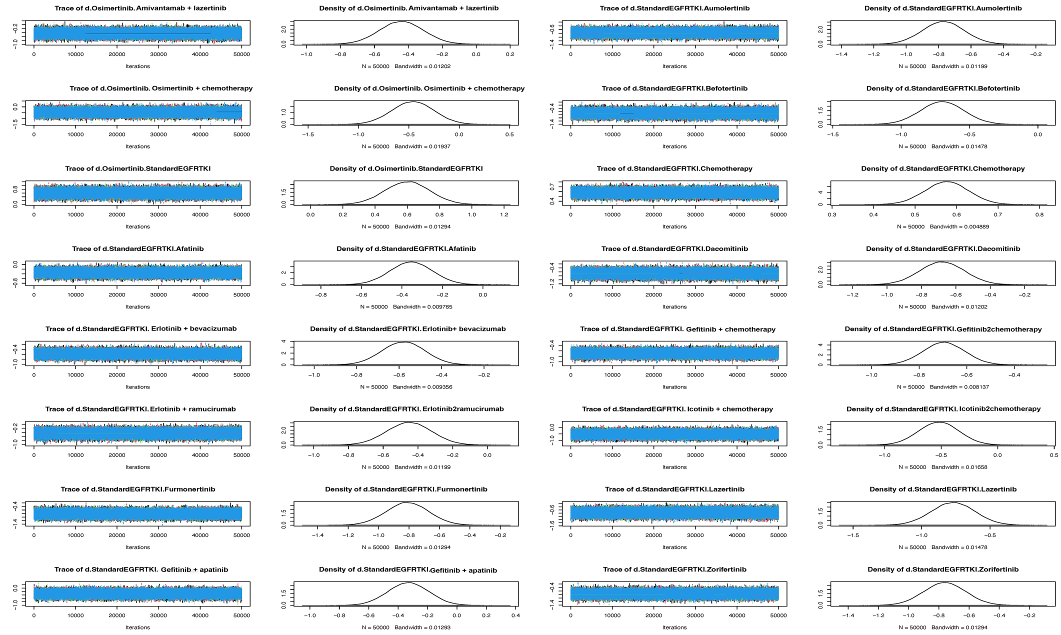

Elderly

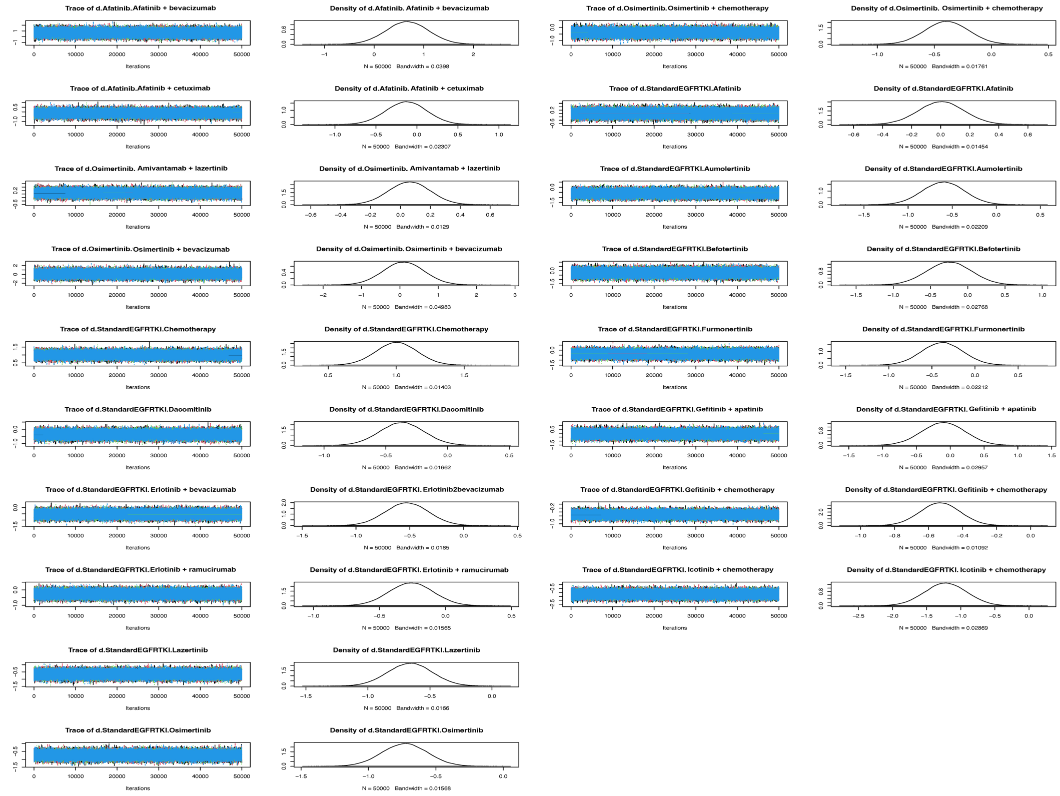

Figure S18 Trace plot for evaluating model convergence of OS (all patients and EGFR mutations)

OS (All patients)

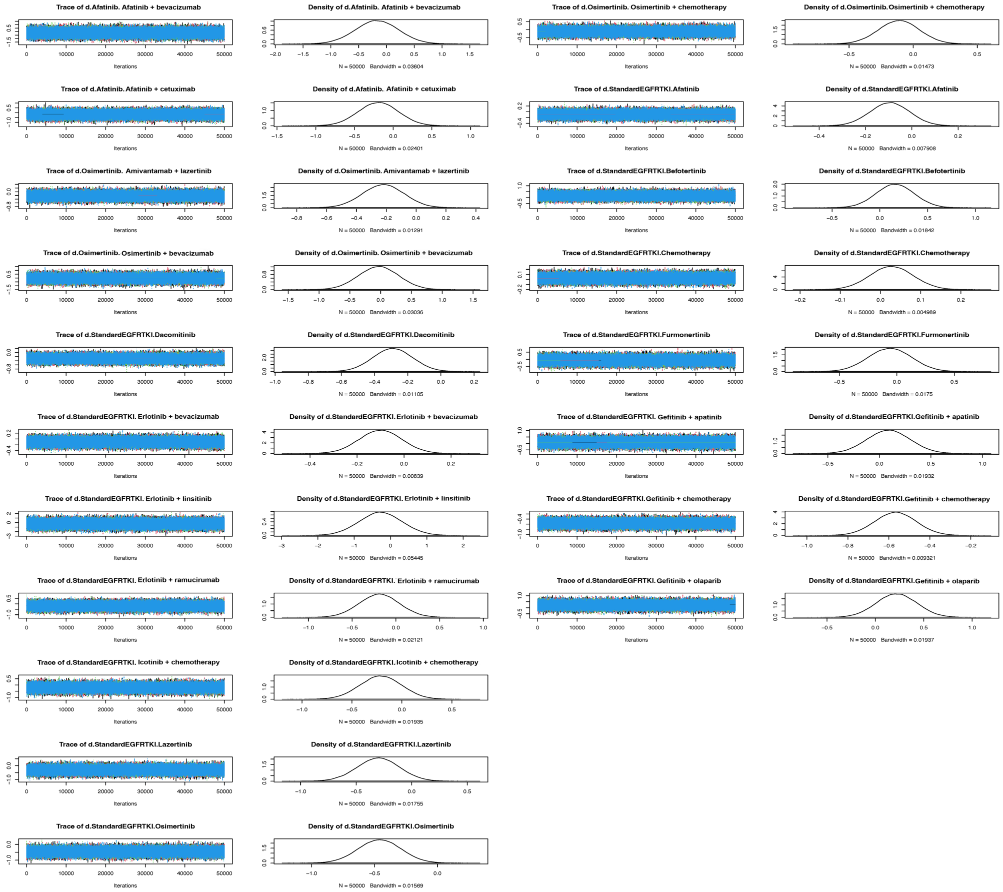

EGFR 19 del

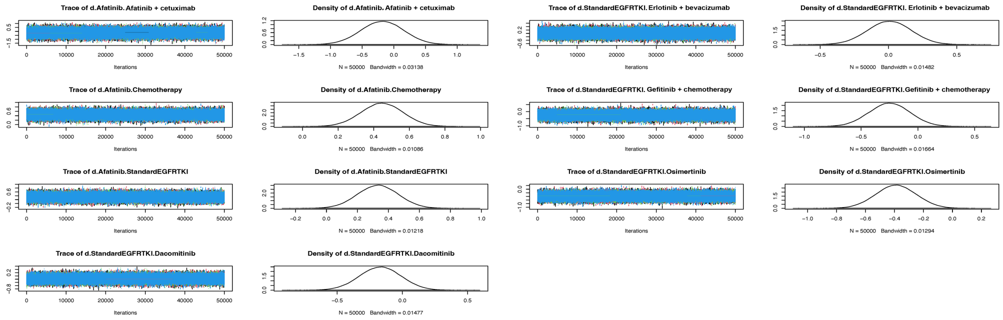

EGFR L858R

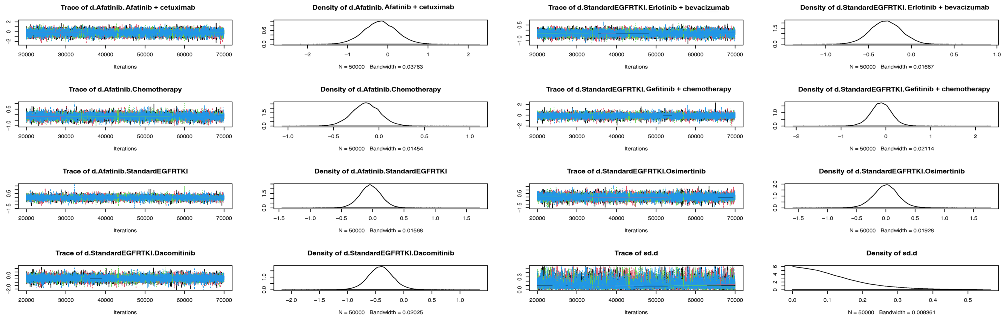

Figure S19 Trace plot for evaluating model convergence of OS (sex, race and age)

Man

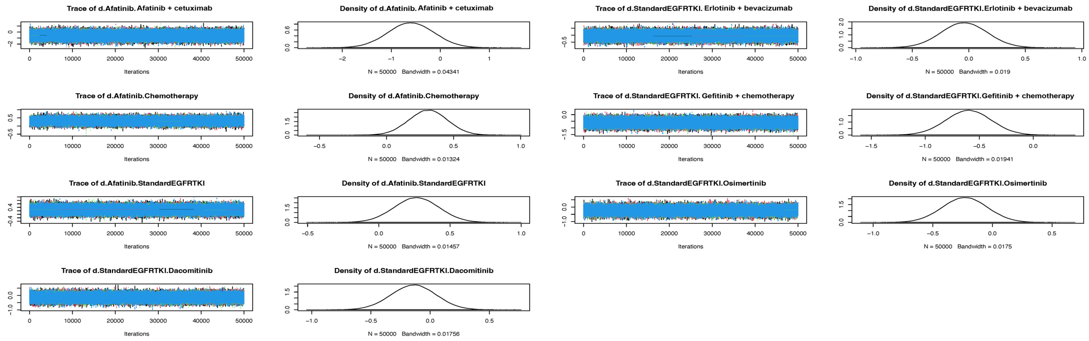

Woman

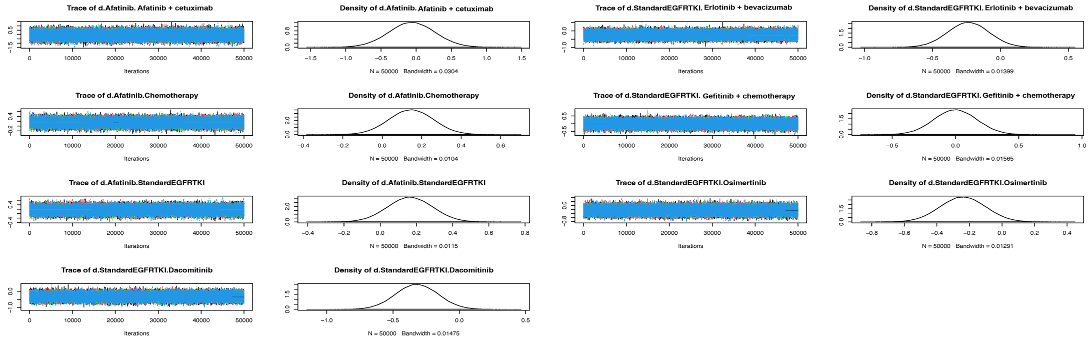

Asian

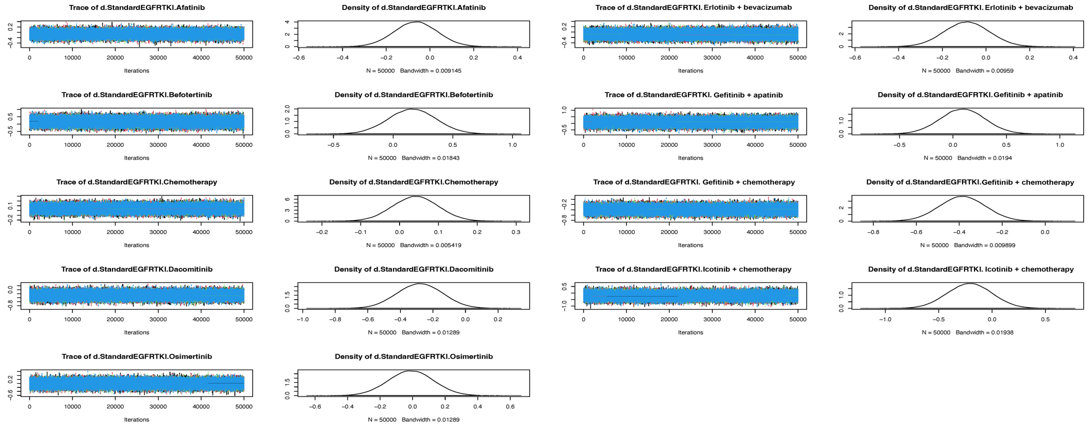

Elderly

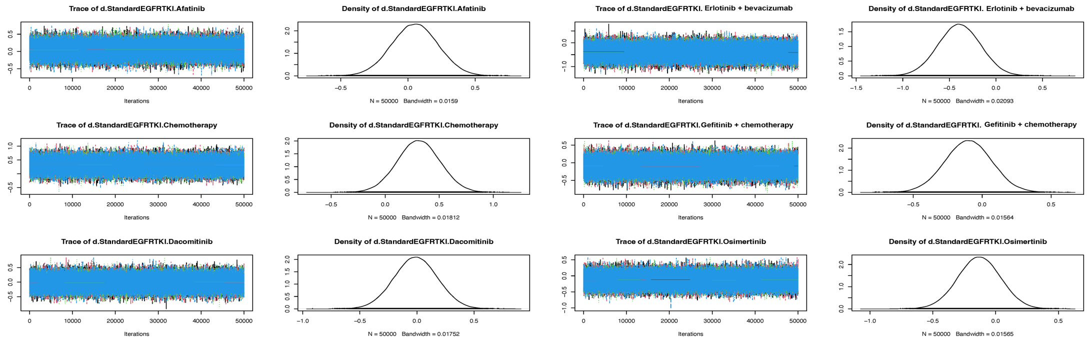

Figure S20 Funnel plot for evaluating publication bias of PFS

A. PFS

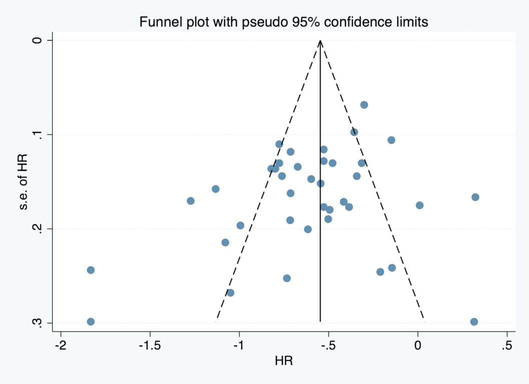

B.OS

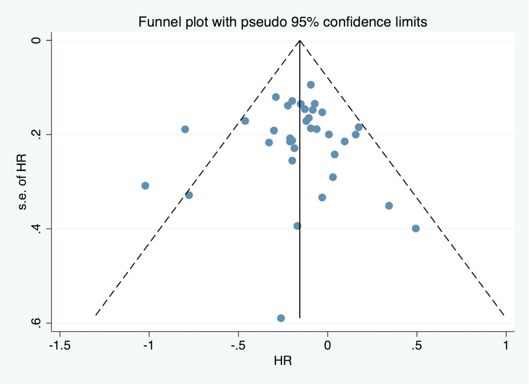

C. PFS(19del)

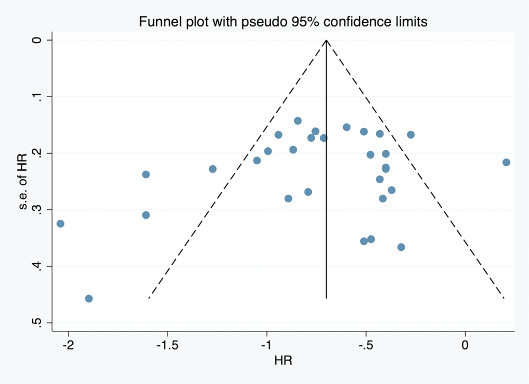

D. PFS(21 L858R)

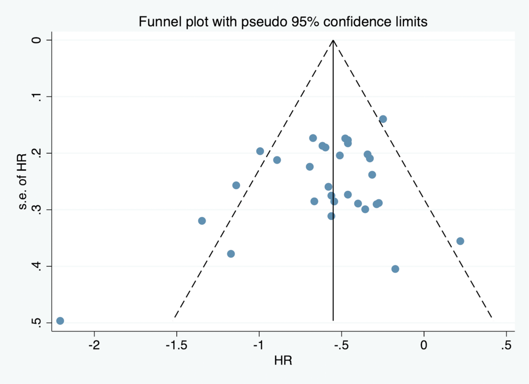

E. PFS(man)

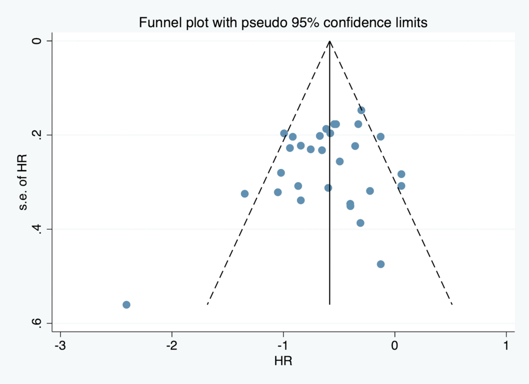

F. PFS(woman)

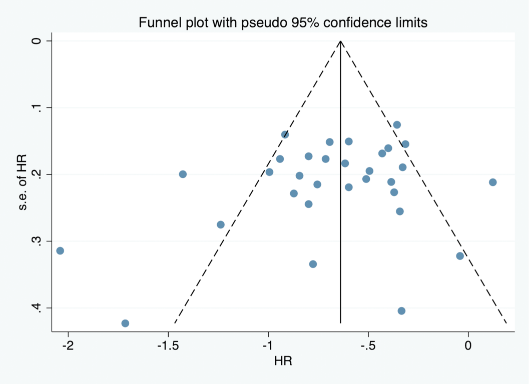

G. PFS(Asian)

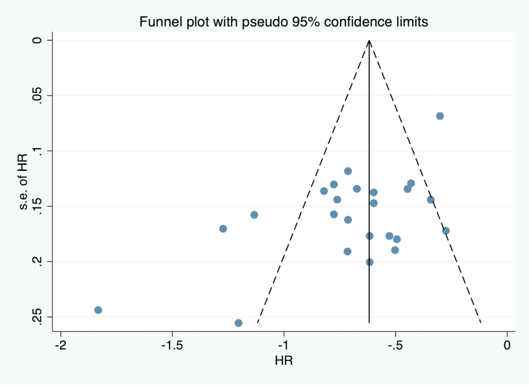

H. PFS(elderly)

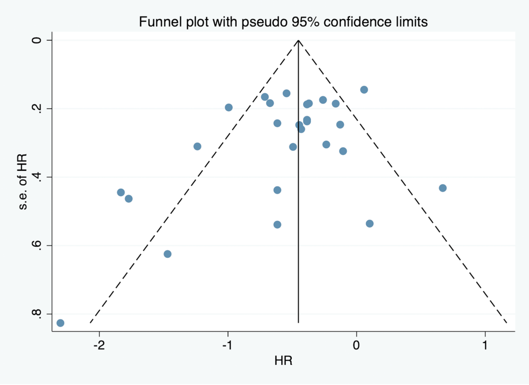

Figure S21 Funnel plot for evaluating publication bias of OS

A. OS(19del)

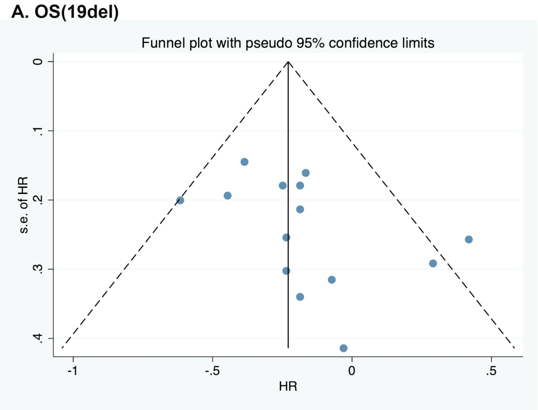

B. OS(21 L858R)

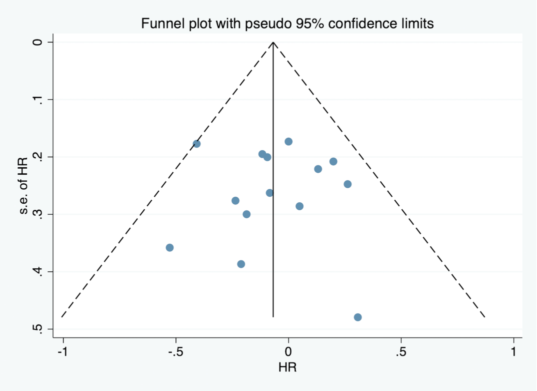

C. OS(man)

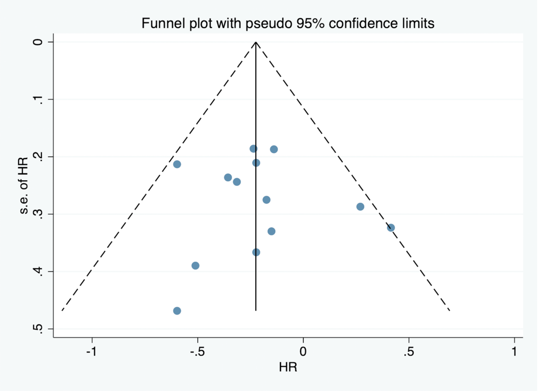

D. OS(woman)

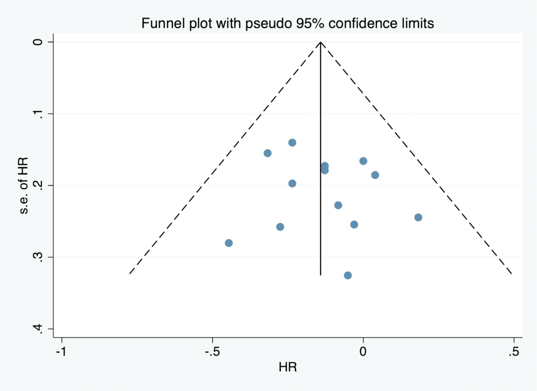

E.OS(Asian)

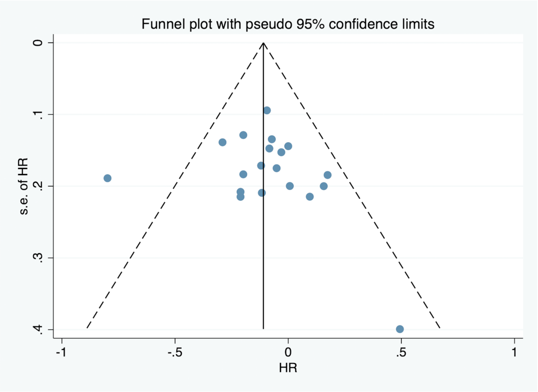

F.OS(elderly)

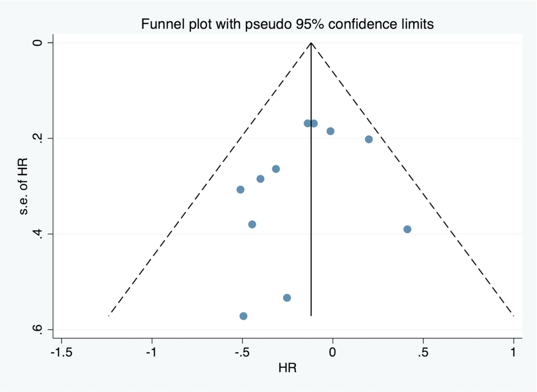

Supplement: Supplementary file 1 — Supplementary Material 1: Table S1. PRISMA extension checklist for this network meta-analysis. Table S2. Search strategy. Figure S1. Network map comparing different treatment outcomes in different groups. Figure S2-S7. Heterogeneity analysis. Figure S8, S9. Inconsistency analysis. Figure S10. Pooled estimates of the network meta-analysis. Figure S11, S12. Ranking diagram for network meta-analysis. Figure S13, S14. Brooks-Gelman-Rubin diagnostic plots for evaluating model convergence. Figure S15-S20. Trace plot for evaluating model convergence. Figure S21, S22. Funnel plot for evaluating publication bias. [file 12885_2025_15236_MOESM1_ESM.pdf]
