# Supplementary figures and images for: Assessing first-line treatment for advanced EGFR-mutated NSCLC in diverse clinicopathological subgroups: a systematic review and network meta-analysis
Source: BMC Cancer. 2025 Nov 14;25:1767. doi: 10.1186/s12885-025-15236-z (PMC12619336; doi:10.1186/s12885-025-15236-z)

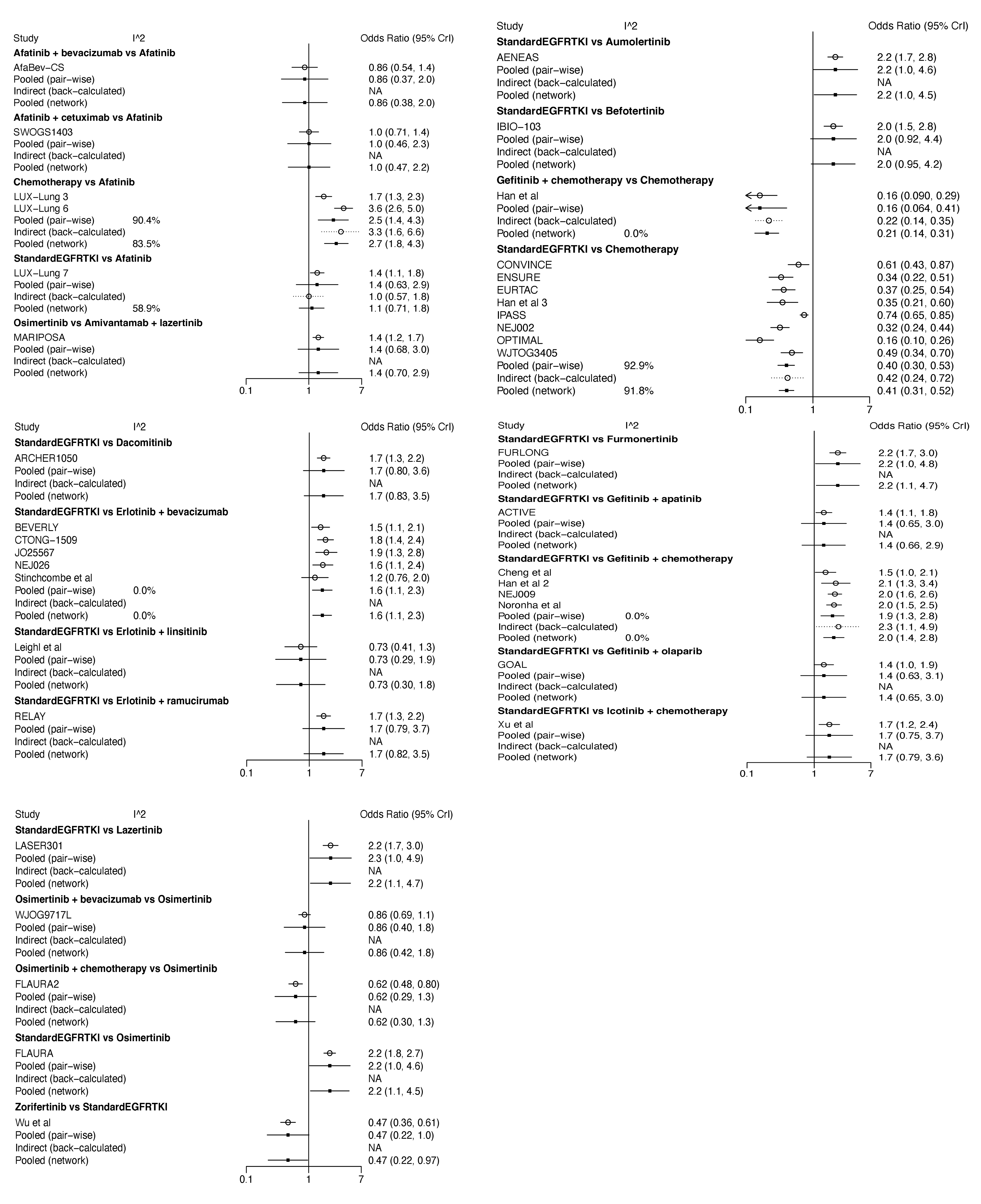

Supplement: Supplementary file 2 — Supplementary Material 2. [file 12885_2025_15236_MOESM2_ESM.tif]

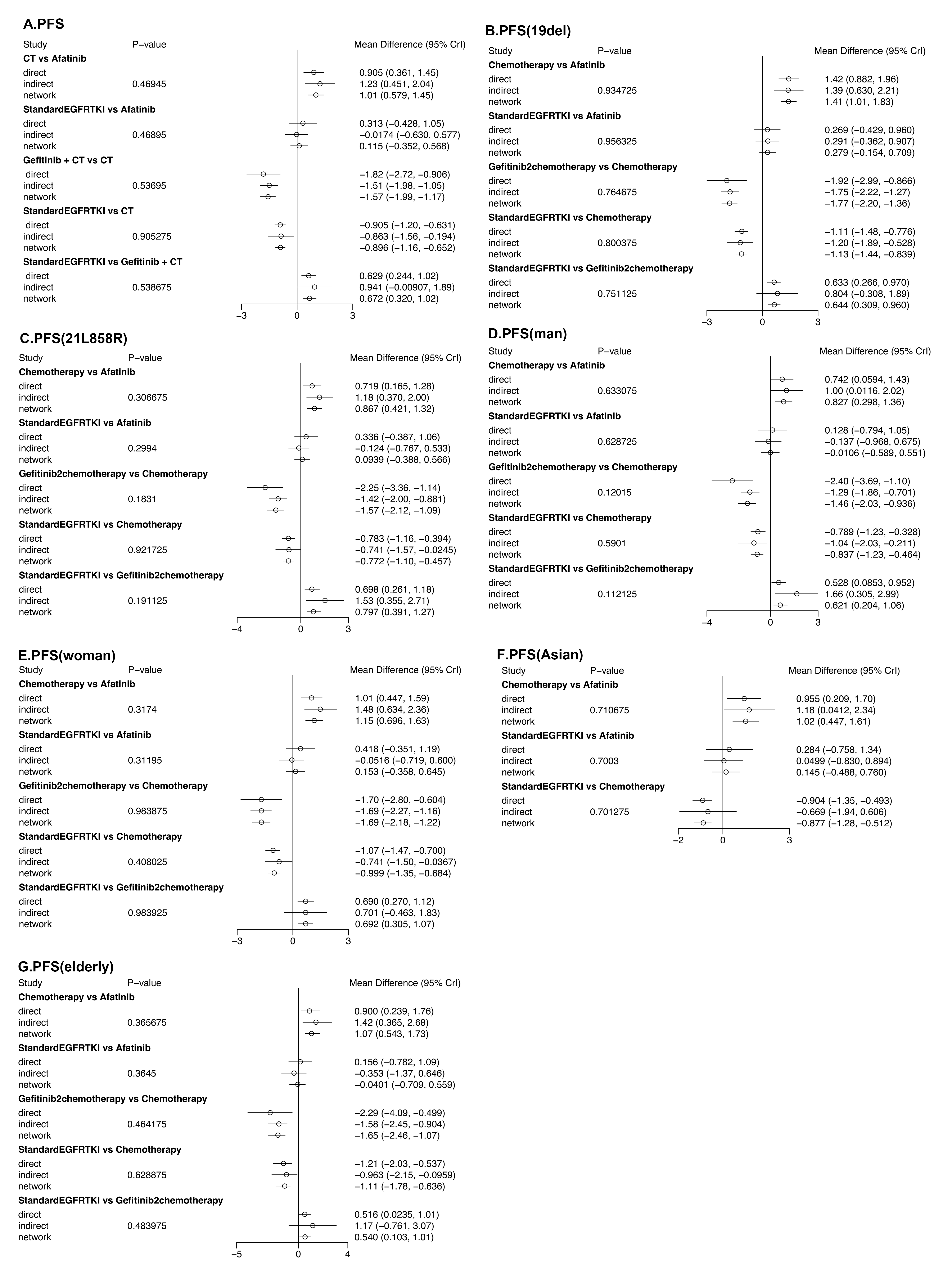

Supplement: Supplementary file 3 — Supplementary Material 3. [file 12885_2025_15236_MOESM3_ESM.tif]

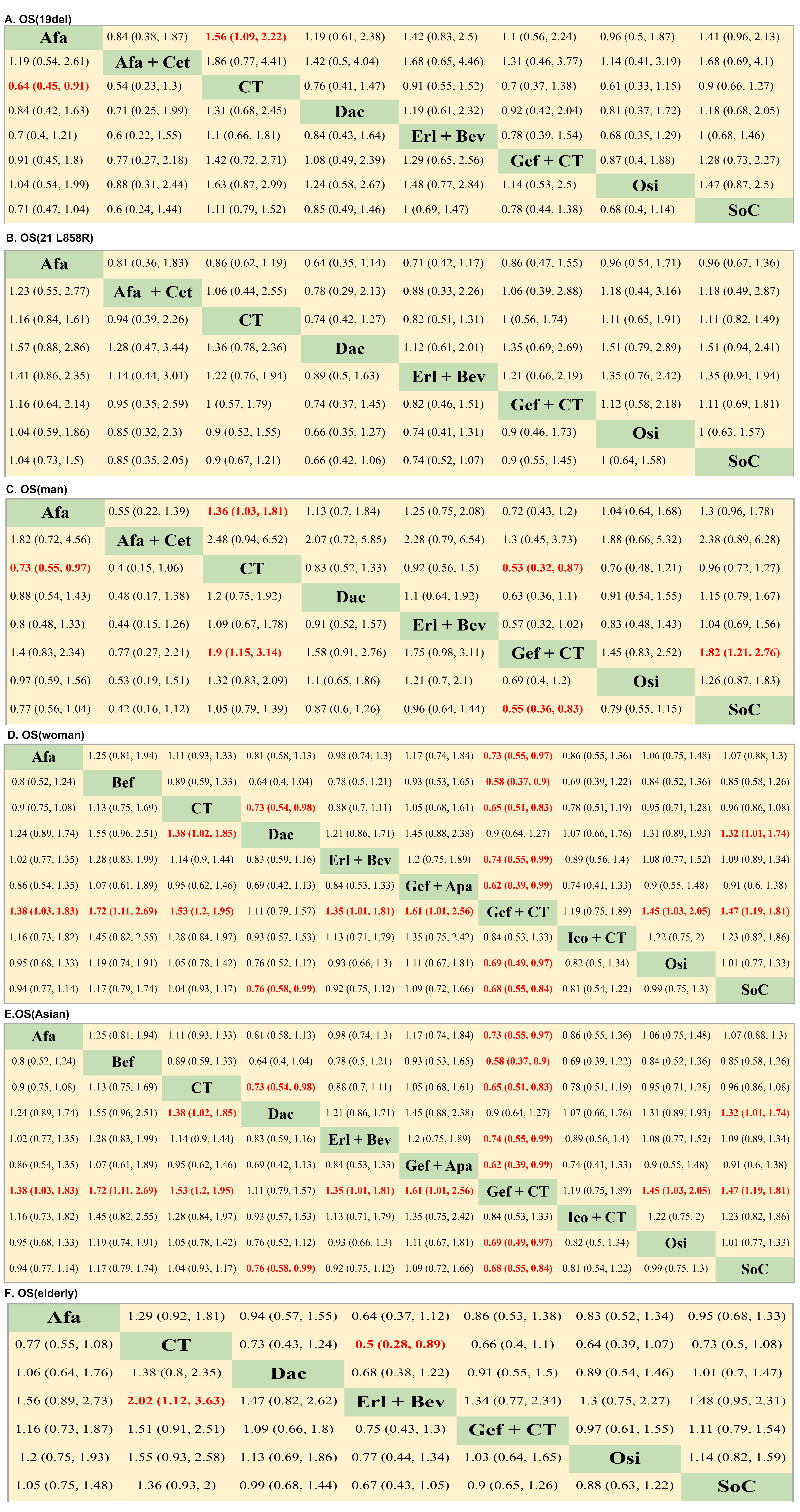

Supplement: Supplementary file 4 — Supplementary Material 4. [file 12885_2025_15236_MOESM4_ESM.tif]

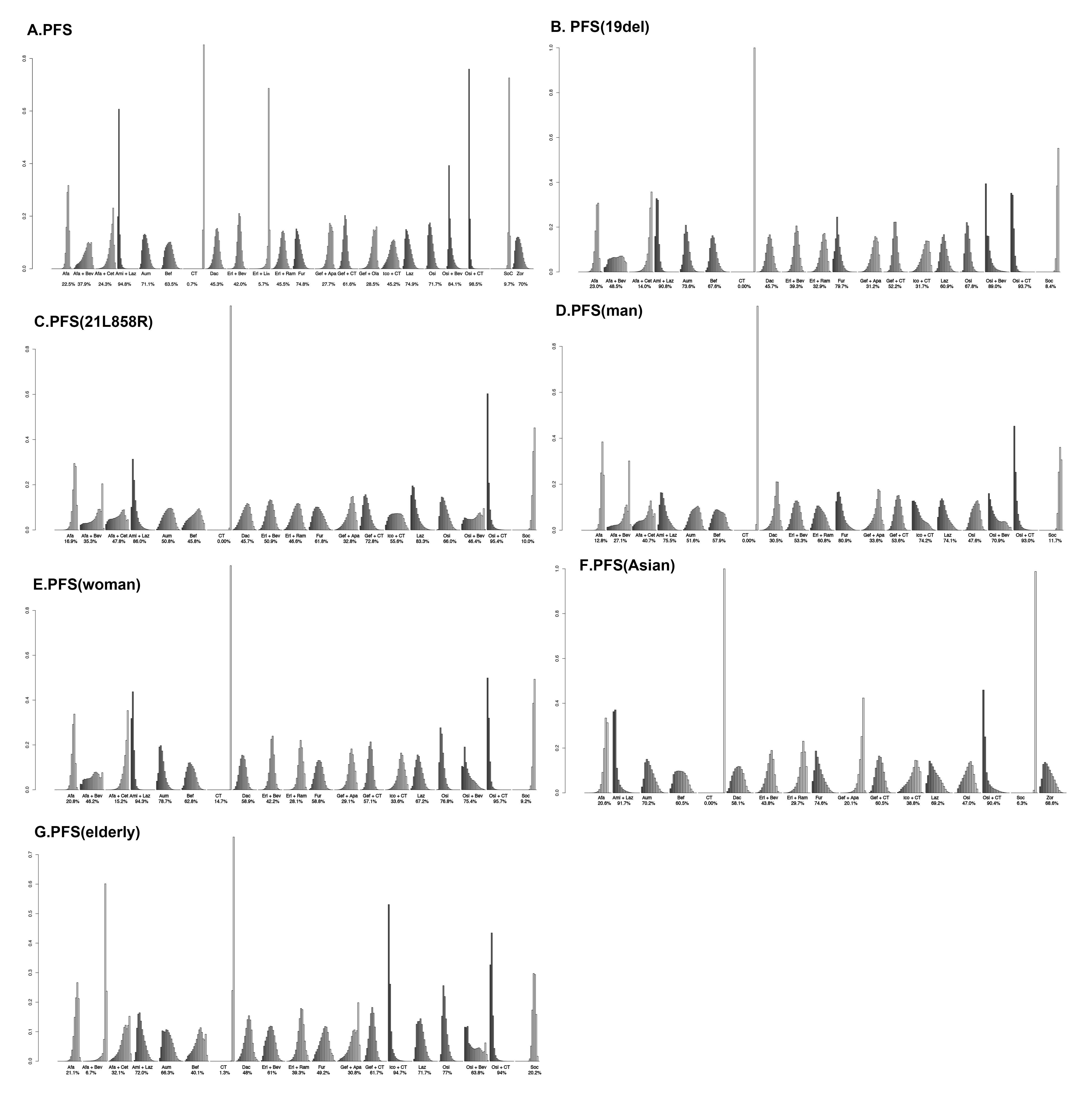

Supplement: Supplementary file 5 — Supplementary Material 5. [file 12885_2025_15236_MOESM5_ESM.tif]

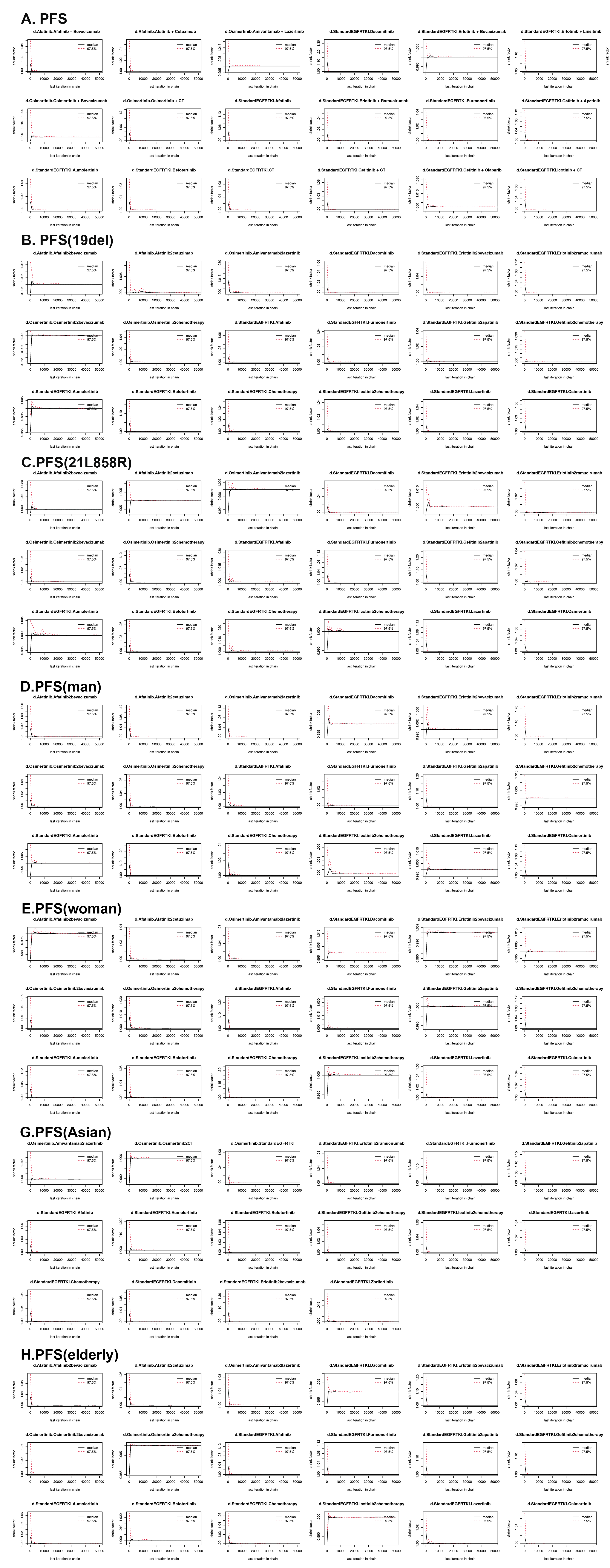

Supplement: Supplementary file 6 — Supplementary Material 6. [file 12885_2025_15236_MOESM6_ESM.tif]

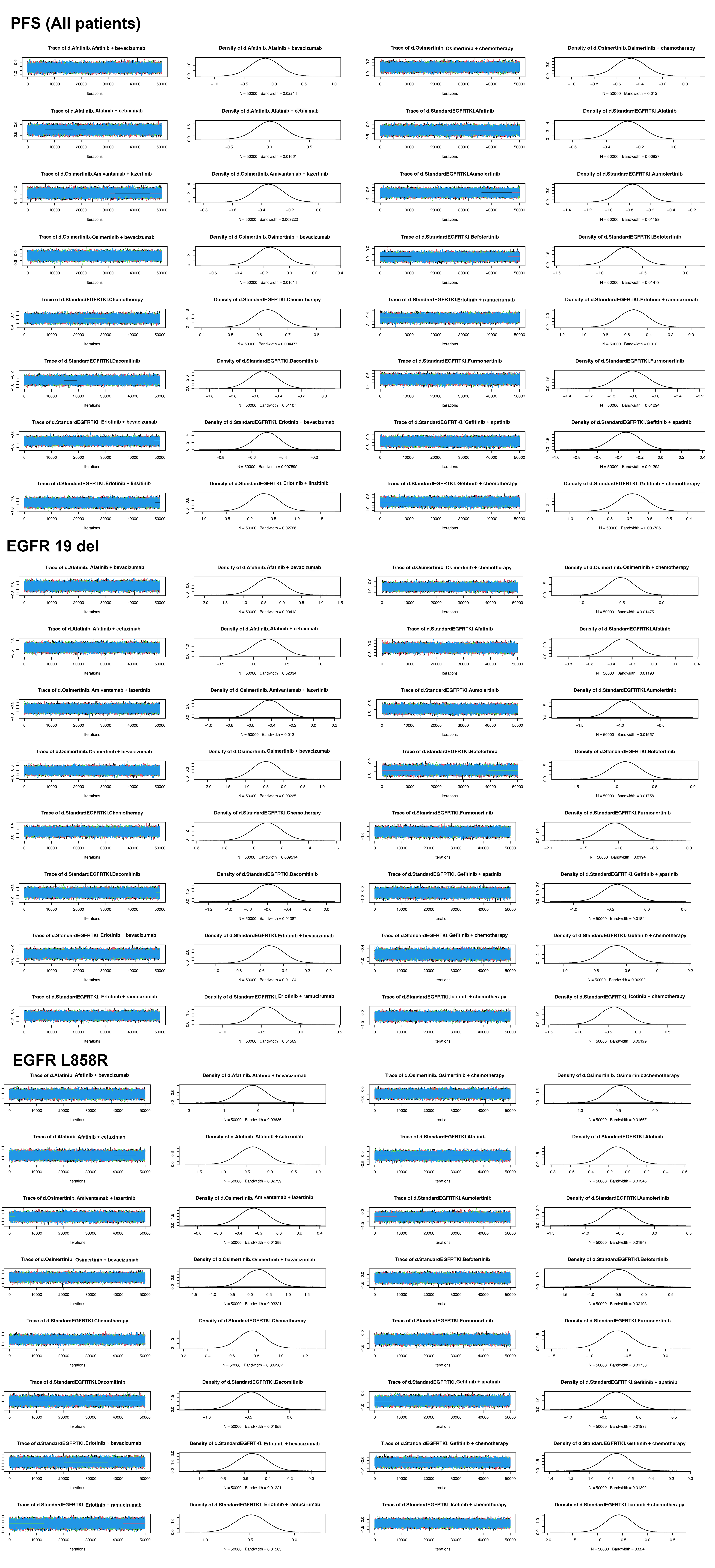

Supplement: Supplementary file 7 — Supplementary Material 7. [file 12885_2025_15236_MOESM7_ESM.tif]

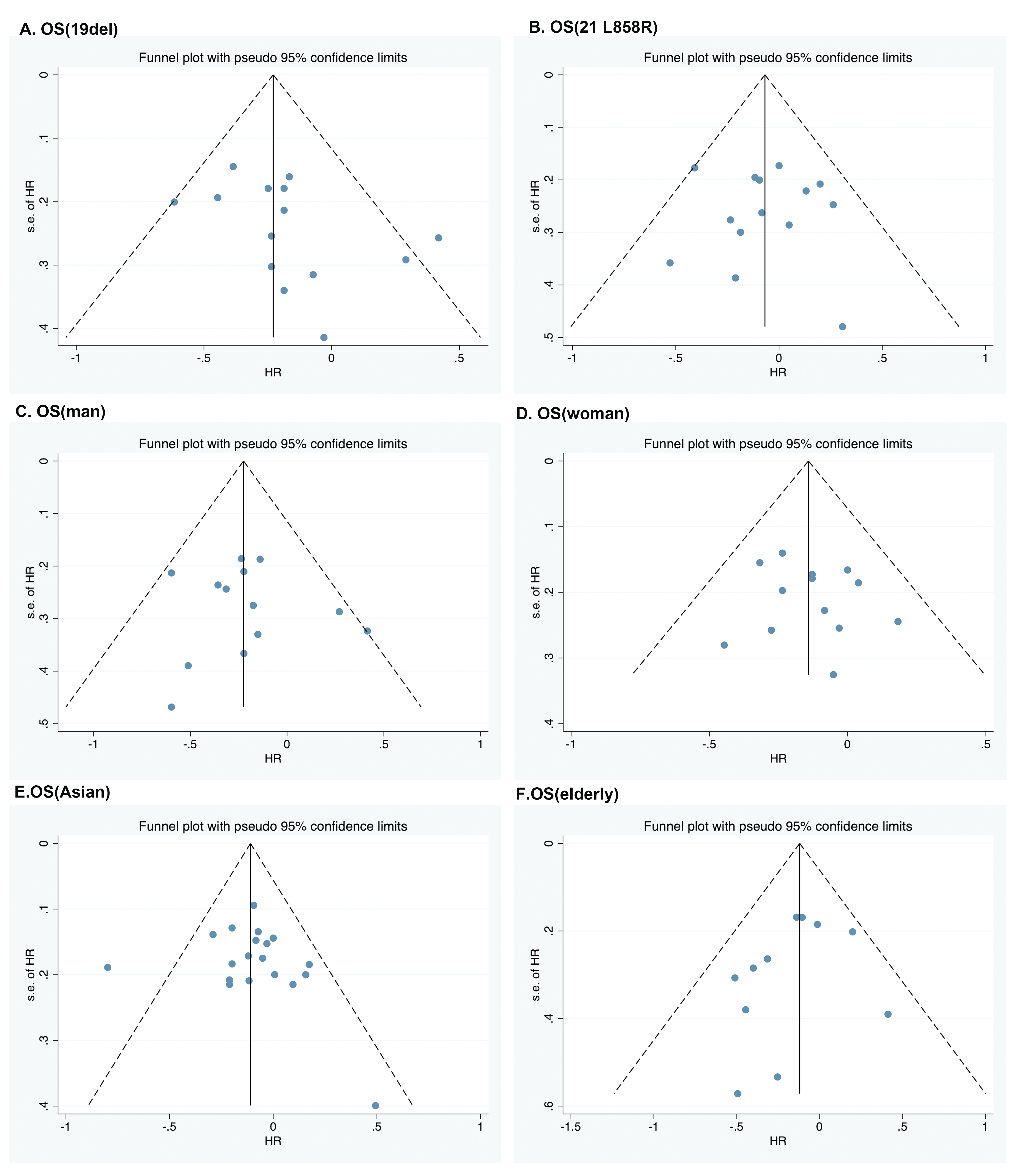

Supplement: Supplementary file 8 — Supplementary Material 8. [file 12885_2025_15236_MOESM8_ESM.tif]
